# Aire Gene Influences the Length of the 3′ UTR of mRNAs in Medullary Thymic Epithelial Cells

**DOI:** 10.3389/fimmu.2020.01039

**Published:** 2020-05-28

**Authors:** Ernna H. Oliveira, Amanda F. Assis, Cesar A. Speck-Hernandez, Max Jordan Duarte, Geraldo A. Passos

**Affiliations:** ^1^Molecular Immunogenetics Group, Department of Genetics, Ribeirão Preto Medical School, University of São Paulo (USP), Ribeirão Preto, Brazil; ^2^State University of Minas Gerais, Passos, Brazil; ^3^Laboratory of Genetics and Molecular Biology, Department of Basic and Oral Biology, School of Dentistry of Ribeirão Preto, USP, Ribeirão Preto, Brazil

**Keywords:** Aire, mTECs, miRNA-mRNA interaction, 3'UTR length, peripheral tissue antigens

## Abstract

Aire is a transcriptional controller in medullary thymic epithelial cells (mTECs) modulating a set of peripheral tissue antigens (PTAs) and non-PTA mRNAs as well as miRNAs. Even miRNAs exerting posttranscriptional control of mRNAs in mTECs, the composition of miRNA-mRNA networks may differ. Under reduction in Aire expression, networks exhibited greater miRNA diversity controlling mRNAs. Variations in the number of 3'UTR binding sites of Aire-dependent mRNAs may represent a crucial factor that influence the miRNA interaction. To test this hypothesis, we analyzed through bioinformatics the length of 3'UTRs of a large set of Aire-dependent mRNAs. The data were obtained from existing RNA-seq of mTECs of wild type or Aire-knockout (KO) mice. We used computational algorithms as FASTQC, STAR and HTSEQ for sequence alignment and counting reads, DESEQ2 for the differential expression, 3USS for the alternative 3'UTRs and TAPAS for the alternative polyadenylation sites. We identified 152 differentially expressed mRNAs between these samples comprising those that encode PTAs as well as transcription regulators. In Aire KO mTECs, most of these mRNAs featured an increase in the length of their 3'UTRs originating additional miRNA binding sites and new miRNA controllers. Results from the *in silico* analysis were statistically significant and the predicted miRNA-mRNA interactions were thermodynamically stable. Even with no *in vivo* or *in vitro* experiments, they were adequate to show that lack of Aire in mTECs might favor the downregulation of PTA mRNAs and transcription regulators via miRNA control. This could unbalance the overall transcriptional activity in mTECs and thus the self-representation.

## Introduction

Autoimmune regulator (Aire) gene encodes a protein that play its role as a transcriptional modulator rather than a classical transcription factor in medullary thymic epithelial cells (mTECs). AIRE protein is considered a non-classical transcription factor due two characteristics found in its mode of action. Firstly, it associates with several protein partners in pushing RNA Pol II on the chromatin, favoring the elongation phase of transcription, and second due to the large set of downstream genes it controls (1–6).

In addition to AIRE, Forebrain embryonic zinc finger-like protein 2 (FEZF2) also play its role in mTECs but as a classical transcription factor (7–9). Most of genes controlled by AIRE encode peripheral tissue antigens (PTAs) that once expressed by mTECs ensure the self-representation in the thymus triggering the elimination of auto-reactive thymocytes though negative selection. Due to the diversity of autoantigens represented, this phenomenon is referred to as promiscuous gene expression (PGE) (3–5, 10–12). AIRE is considered the main controller of PGE/self-tolerance in the thymus and its disruption caused by mutations results in an aggressive autoimmune disease referred to as APECED or APS-1 (13–15).

The meaning of the Aire expression is relative to the immunological tolerance that occurs in the thymus, since the expression of this gene is preferentially in that organ. Other organs such as pancreas, adrenal cortex, testes, lymph nodes, fetal liver and appendix also express Aire, without however knowing its exact function in these organs (6, 16–19).

The human AIRE gene maps on chromosome 21q22.3 and in the mouse on chromosome 10 position 39.72 cM and the primary structure of Aire gene suggests that the distribution of its domains encodes a protein that interacts with the chromatin and regulates transcriptional processes (2, 20–27).

Nevertheless, AIRE protein does not act as a classical transcription factor, which binds directly to gene promoters' consensus sequences. Instead of, AIRE forms a multimolecular complex in which participates the so termed AIRE-partners (CBPBP, SIRT-1, DNA-PK, PARP-1, DNA-TOP2a, BRD4, P-TEFb) that work together in the releasing of stalled RNA-Pol II in the chromatin (4, 5, 28). Not yet fully resolved if acetylation of lysine residues of AIRE by CREB-binding protein (CBPBP) stabilizes intracellular localization of AIRE protein (29, 30), but group-III histone-deacetylase SIRT-1 preserves AIRE-dependent PGE by deacetylation of such residues (31, 32).

Of note, the DNA-topoisomerase (DNA-TOP2a) protein, which is a strong AIRE partner acts on the DNA topology by removing positive and negative supercoils when it causes transient DNA breaks. DNA breaks result in a local relaxation of chromatin that facilitates the initiation and post-initiation of gene transcription (6, 33) and attracts DNA repair elements such as DNA-PK and PARP1 (6).

However, it is the interaction with PTEFb that resolves that AIRE play its role in the post-initiation phase of transcription (34). As previously discussed (6), gene transcription in eukaryotic cells is annulled if PTEFb does not proceed with the elongation and splicing of pre-mRNA into mature mRNA and subsequent release of stalled RNA Pol II (35). In turn, bromodomain-containing protein 4 (BRD4) interacts with PTEFb being that balanced phosphorylation and acetylation of AIRE are needed to preserve such interaction (36).

Evidence suggests that AIRE and its partners bind to chromatin super-enhancers, which include the transcription start sites of most Aire-dependent genes (37). This multimolecular complex enables the transcription elongation step of a myriad of genes including those encoding autoantigens (4, 6, 28).

AIRE also associates to proteins involved with pre-mRNA processing (1), alternative splicing (38) and polyadenylation factors (HNRNPL/F, SFRS1, and CLP1) (39). It is plausible that this might be directly or indirectly related to the processing of Aire-dependent mRNAs in mTECs.

By means of classical gene knockout (KO) (40–42) or gene KO through Crispr-Cas9 (43) it was demonstrated that Aire affects the abundance of mRNA isoforms in mTECs. Previously we observed that miRNAs exert posttranscriptional effect on Aire-dependent mRNAs (44). However, this action is not uniform and a greater set of miRNAs exert their effect on mRNAs when Aire is down-regulated in mTECs (45).

These findings prompted us to investigate whether Aire could affect isoforms of mRNAs involving the length of 3'UTR end as we have previously suspected (3). Whether this process exists among the Aire-dependent mRNAs, this may be influencing the control of the PGE and consequently of the self-representation in the thymus. Accordingly, the aim of this study was to evaluate whether Aire affects the length of the 3'UTR region of mRNAs it controls.

To test this we analyzed the existing RNA-seq data of mTECs isolated from Aire wild-type or Aire-knockout (KO) mice (41) identifying and quantifying those mRNAs differentially expressed between these two samples and that showed changes in the length of the 3'UTR region and thus in the miRNA binding sites.

## Materials and Methods

### RNA-Seq Data Analysis

We obtained the sequences from raw RNA-seq data deposited in the Gene Expression Omnibus GEO repository (https://www.ncbi.nlm.nih.gov/geo/) and available under accession number GSE65617 (41), which consisted of RNA-seq of CD45^−^ EPCAM^+^ sorted as cTECs (Ly51^+^ UEA1^−^), Ly51^−^ UEA^+^ CD80^lo^ MHC-II^lo^ sorted as mTEC^lo^, Ly51^−^ UEA^+^ CD80^hi^ MHC-II^hi^ sorted as mTEC^hi^. The analyzed sequences were from mTEC^hi^ cells (Ly51^−^ UEA^+^ CD80 ^hi^ MHC II ^hi^) isolated of wild type (WT) or Aire knockout (KO) C57BL/6 mice. Each biological replicate (*n* = 3 for each cell type) consisted of a pool of mTECs (FACS sorted) of four isogenic animals (two males and two females from the same litter) WT or KO. Sequencing was performed on an Illumina HiSeq 2000 RNA-seq platform with paired-end libraries with fragments of 100 bp (2 × 100 bp).

Analyses of RNA sequences were performed according to the following pipeline adopted for this study. The Fastq sequences provided by GEO were initially checked for quality through the FASTQC program (http://www.bioinformatics.babraham.ac.uk/projects/fastqc/). Then, the Fastq sequences were aligned against the *Mus musculus* reference genome (mm10) (Mouse Genome Reference Consortium https://www.ncbi.nlm.nih.gov/grc/mouse) through the Spliced Transcripts Alignment to a Reference (STAR 2.5) program (https://github.com/alexdobin/STAR) (46), which outputs a BAM file containing both the sequence and its genomic coordinate.

Together with a General Transfer Format (GTF) the file containing all the genomic annotations were used to determine the total number of reads per transcript using the HTSeq Count program (https://htseq.readthedocs.io/en/release_0.11.1/) (27).

Information about genome assembly and gene annotation corresponds to the mm10 reference genome that was submitted by the Genome Reference Consortium (mouse genome reference GRCm38). The complete description of this annotation as for example, base pair counts, gene counts among other information, can be found available at (m.ensembl.org/Mus_musculus/Info/Annotation).

For each sample analyzed, we obtained a list of genes with their respective counts, which were analyzed to determine the genes differentially expressed by using the DESEq2 package (https://bioconductor.org/) (47), which is carried out through the platform R (https://www.r-project.org/). A heat-map was constructed in order to hierarchically cluster samples and mRNAs based on Euclidean distance and the complete linkage method.

In this work, genes with a *p* < 0.05, Benjamini-Hochberg FDR correction and fold change ≥ 1.5, were considered as differentially expressed.

### mRNA 3'UTR and Alternative Polyadenylation Site (APA) Analysis

For evaluation of changes in the 3'UTR sequences of mRNAs expressed in WT or Aire KO mTEC cells, we used the 3'UTR Sequence Seeker (3USS) (https://bio.tools/3uss) program. The 3USS program automatically identifies alternative 3'UTR sequences based on alternative polyadenylation sites (APA) obtained from actual RNA-seq data (long or short sequences) in relation to the mouse reference genome and transcriptome annotated in the UCSC (https://genome.ucsc.edu), NCBI (http://www.ncbi.nlm.nih.gov/), Ensembl (http://www.ensembl.org/index.html) and Gencode (http://www.gencodegenes.org/) databases. In addition, this program attempts to identify new 3'UTR sequences, which have not yet been annotated in public databases (48).

We then classify the 3'UTR alternative sequences into two categories, Aire-dependent or Aire-independent considering the differentially expressed genes between WT and Aire KO mTECs.

Next, we focused on the 3'UTR alternative sequences of Aire-dependent peripheral tissue antigens (PTAs) mRNAs. The identification of PTA mRNAs was done based on the classification of Sansom (40) that use both the HSGeneAtlas (https://dintor.eurac.edu/doc/doc/HSGeneAtlas.html) and the mTEC transcriptome obtained by these authors as a parameter. Alignment between the reference sequences and 3'UTR alternative sequences of mRNAs encoding Aire-dependent PTAs was done through the BLAST program (http://blast.ncbi.nlm.nih.gov/Blast.cgi?CMD=Web&PAGE_TYPE=BlastHome) (49).

In addition, we use the TAPAS tool (https://github.com/arefeen/TAPAS) for the determination of the number of APA sites from RNA-seq data, which works with two or more APA sites or with APA sites located prior to the last exon of a RNA target sequence (50). This tool compares RNA-seq data of pairs of biological samples that in this study were samples from Aire WT or Aire KO mTECs. The tool identify those differentially expressed APA sites as well as mRNAs that contain 3'UTRs with shortening/ lengthening events.

### Interactions Between miRNAs and Aire-dependent PTA mRNAs

In order to identify those interactions between miRNAs and PTA mRNAs that were generated by alternative increase in the 3′UTR sequence, the miRDB database (http://www.mirdb.org/) was used. We used as a criterion to include a given miRNA in the interaction networks, only those that in the mirdb.org database presented a target score between 90 and 100. This database is connected to MirTarget tool, which allows miRNA target prediction and functional annotations. To assess whether the interactions from this study had already been predicted, we performed a survey of those miRNAs that expressively interact with mRNAs that encode PTAs using the following public databases, microT-CDS (http://diana.imis.athena-innovation.gr/DianaTools/index.php?r=microT_CDS/index), microRNA.org (http://www.microrna.org/microrna/home.do), miRDB (http://mirdb.org/), miRWalk (http://zmf.umm.uni-heidelberg.de/apps/zmf/mirwalk2/), Pictar5 (https://omictools.com/pictar-tool), Pita (https://omictools.com/pita-tool), RNA22 (https://cm.jefferson.edu/rna22/) and Targetscan (http://www.targetscan.org/vert_72/). New interactions were those identified in this study, but not predicted and deposited in any of these databases.

### *In silico* Validation of miRNA-mRNA Interactions

The new and predicted miRNA-mRNA interactions were validated using the RNA-Hybrid algorithm (http://bibiserv.techfak.uni-bielefeld.de/rnahybrid) (51). It is a bioinformatics tool that analyzes the secondary structure, and determines the most favorable hybridization site between a given miRNA and its target mRNA. The methodology involves a dynamic programming algorithm that calculates the location for the most energy-efficient hybridization. This methodology is based on the thermodynamic property of miRNA-mRNA interaction, in which the RNA double strand is more stable, that is, the miRNA-mRNA binding is stronger when the energy is lower. The interaction force is determined by the quantification of the minimum free energy (mfe) normalized by the length of the target mRNA. In this study, we selected interactions with mfe ≤ −20 kcal / mol, which are considered thermodynamically stable.

### Functional Enrichment of Non-PTA Aire-dependent mRNAs

To better understand the functional annotation of Aire-dependent mRNAs other than those encoding PTAs, but that featured alterations in the length of their 3′UTRs, we use the DAVID (david.ncifcrf.gov/) considering a *P*-value EASE score ≤ 0.1 to examine the significance of gene-term enrichment with a modified Fisher's exact test. The false discovery rate (FDR) under certain rate (e.g., ≤ 0.05) provided by DAVID is calculated on the basis of the multiple testing correction as Bonferroni and Benjamini methods.

## Results

### 3'UTR Shortening / Lengthening Events Among the Differentially Expressed mRNAs

**Figure 1:**
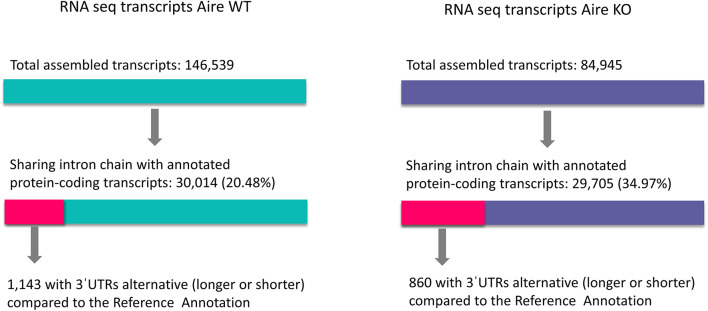
Shortening or lengthening of 3'UTR in mRNAs. Changes in 3'UTR of mRNAs expressed by Aire wild type or Aire KO mTECs were quantified using the *Mus musculus* reference genome, version GRCm38/mm10.

For identification of possible changes in 3′UTR sequences of mRNAs expressed in mTEC cells, transcripts assembled from the WT (146,539 transcripts) or KO mTECs (84,945 transcripts) were used as input to the 3USS program. This program automatically identified alternative 3′UTR sequences, using as reference the *Mus musculus* genome, version GRCm38/mm10. The set of transcripts assembled from the WT mTECs comprised 30,014 and the set from KO mTECs comprised 29,705 protein encoding mRNAs. Of the mRNAs expressed by the WT mTECs, 1,143 showed changes in their 3'UTRs (shortening / lengthening) and of the mRNAs expressed by the KO mTECs, 830 showed changes in their 3'UTRs (Figure 1).

**Figure 2:**
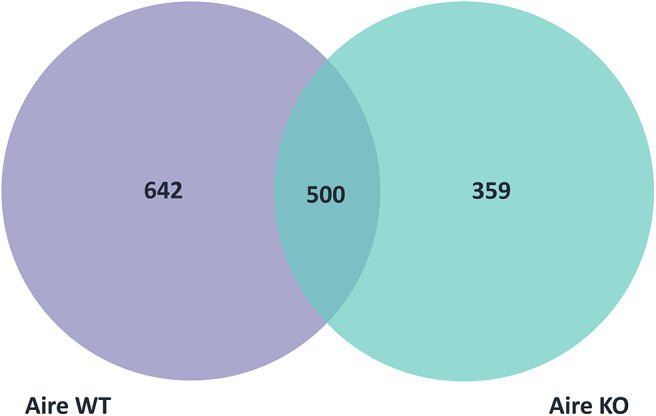
Venn diagram showing the three categories of mTEC transcripts with short or long 3'UTRs: 642 transcripts were specific of Aire wild type, 359 were specific of Aire KO and 500 were common to Aire wild type and Aire KO mTECs.

**Figure 3:**
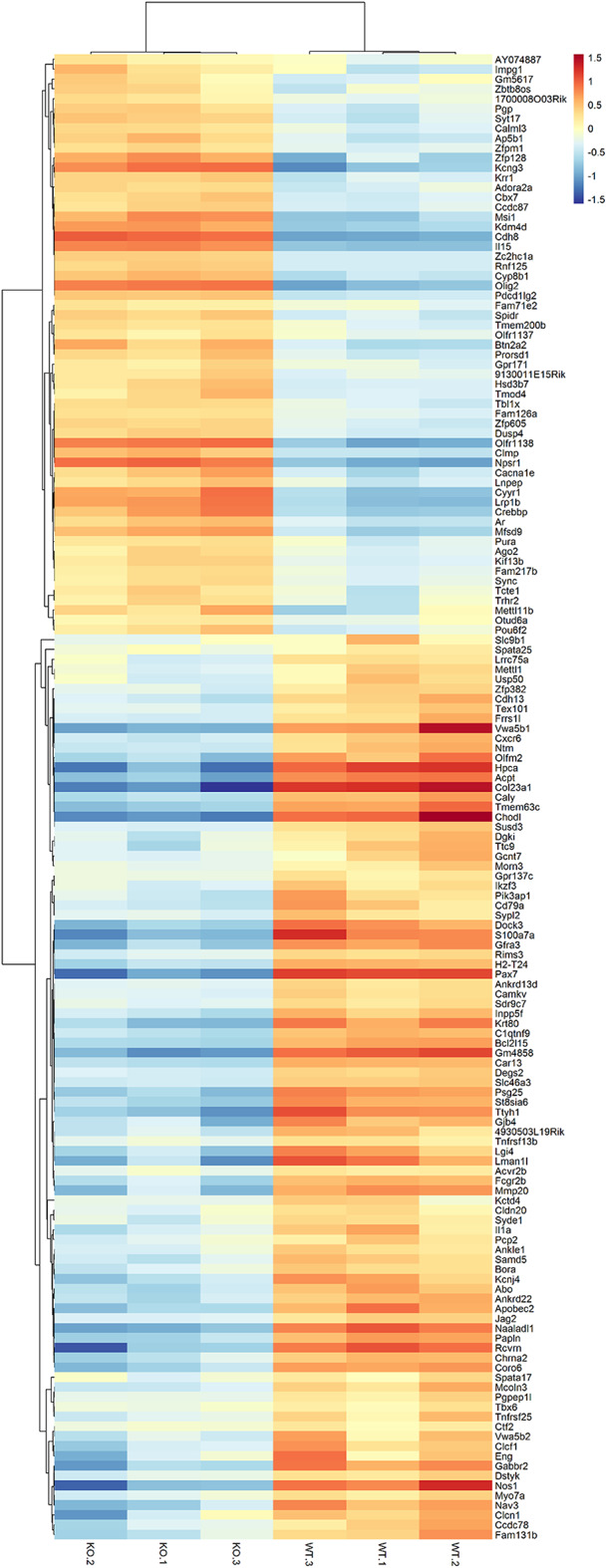
Heat-map showing the large-scale expression profiling of Aire-dependent transcripts when comparing Aire wild-type vs. Aire KO mTECs. Unsupervised heat-maps and dendrograms were constructed using R platform. Red, upregulated mRNAs, blue, downregulated mRNAs (Fold-change FC ≥ 1.5, false-discovery rate, FDR ≤ 0.05, Pearson correlation metrics, 75 percentile).

Then, WT or KO mTEC transcripts, which had the modified 3′UTRs sequences (short or long 3′UTRs) were regrouped into three categories: (1) those specific of WT mTECs (642 transcripts); (2) those specific of KO mTECs (359 transcripts); and (3) common between WT and KO mTECs (500 transcripts) (Figure 2). From these categories, we selected only the 359 KO specific transcripts of which 152 were differentially expressed when comparing WT vs. KO mTECs. Among these transcripts, 41 represent PTA mRNAs and 111 non-PTA mRNAs. This set was considered as differentially expressed Aire-dependent transcripts, which were hierarchically clustered and selected for further analysis (Figure 3, Table 1 and Supplementary Table 1). For further information about gene symbol, full name, chromosomal localization and biological processes, see Supplementary Table 2).

**Table 1:** Differentially expressed PTA and non-PTA mRNAs that featured alternative 3'UTRs.

	**mRNA symbol**		
1700008O03Rik	Crebbp	Krt80	Sdr9c7
4930503L19Rik	Cxcr6	Lgi4	Slc46a3
9130011E15Rik	Cyp8b1	Lman1l	Slc9b1
Abo	Cyyr1	Lnpep	Spata17
Acpt	Degs2	Lrp1b	Spata25
Acvr2b	Dgki	Lrrc75a	Spidr
Adora2a	Dock3	Mcoln3	St8sia6
Ago2	Dstyk	Mettl1	Susd3
Ankle1	Dusp4	Mettl11b	Syde1
Ankrd13d	Eng	Mfsd9	Sync
Ankrd22	Fam126a	Mmp20	Sypl2
Ap5b1	Fam131b	Morn3	Syt17
Apobec2	Fam217b	Msi1	Tbl1x
Ar	Fam71e2	Myo7a	Tbx6
AY074887	Fcgr2b	Naaladl1	Tcte1
Bcl2l15	Frrs1l	Nav3	Tex101
Bora	Gabbr2	Nos1	Tmem200b
Btn2a2	Gcnt7	Npsr1	Tmem63c
C1qtnf9	Gfra3	Ntm	Tmod4
Cacna1e	Gjb4	Olfm2	Tnfrsf13b
Calml3	Gm4858	Olfr1137	Tnfrsf25
Caly	Gm5617	Olfr1138	Trhr2
Camkv	Gpr135	Olig2	Ttc9
Car13	Gpr137c	Otud6a	Ttyh1
Cbx7	Gpr171	Papln	Usp50
Ccdc78	H2-T24	Pax7	Vwa5b1
Ccdc87	Hpca	Pcp2	Vwa5b2
Cd79a	Hsd3b7	Pdcd1lg2	Zbtb8os
Cdh13	Ikzf3	Pgp	Zc2hc1a
Cdh8	Il15	Pgpep1l	Zfp128
Chodl	Il1a	Pik3ap1	Zfp382
Chrna2	Impg1	Pou6f2	Zfp605
Clcf1	Inpp5f	Prorsd1	Zfpm1
Clcn1	Jag2	Psg25	
Cldn20	Kcng3	Pura	
Clmp	Kcnj4	Rcvrn	
Col23a1	Kctd4	Rims3	
Coro6	Kdm4d	Rnf125	
Ctf2	Kif13b	S100a7a	
	Krr1	Samd5	

Next, we focused on the mRNAs that encode PTAs to evaluate the type of change that occurred in their 3'UTRs. We observed that most of them (39/41) showed increase in their 3'UTR sequences, ranging from +651 nt (Cxcr6 mRNA) to +7,724 nt (Rcvrn mRNA). Regarding the shortening, only Ttc9 and Nos1 PTA mRNAs showed reduction of their of the 3'UTR sequences corresponding to −2.004 and −2.136 nucleotide, respectively. Table 2 summarizes the nucleotide increase counts among transcripts analyzed.

**Table 2:** Identification of Aire-dependent PTA mRNAs that featured increased (+) or reduced (–) 3'UTRs in Aire KO mTECs, in comparison to the *Mus musculus* (mm10) reference genome.

**mRNA symbol**	**Reference sequence (NCBI)**	**3'UTR increase/reduction (nucleotides)**
Ankrd22	NM_024204	+1,392
Apobec2	NM_009694	+1,400
Abo	NM_001290444	+2,461
Bcl2l15	NM_001142960	+1,947
C1qtnf9	NM_183175	+755
Caly	NM_001190386	+797
Chodl	NM_139134	+806
Clcf1	NM_019952	+879
Clcn1	NM_013491	+1,218
Col23a1	NM_153393	+781
Coro6	NM_139128	+929
Cxcr6	NM_030712	+651
Dock3	NM_153413	+658
Fam131b	NM_001286584	+1,664
Gfra3	NM_010280	+1,501
Gjb4	NM_008127	+1,181
H2-T24	NM_008207	+2,897
Hpca	NM_001286083	+753
Kcnj4	NM_008427	+2,385
Krt80	NM_028770	+1,251
Lgi4	NM_144556	+2,136
Mcoln3	NM_134160	+748
Morn3	NM_029112	+679
Naaladl1	NM_001009546	+3,014
Nav3	NM_001081035	+1,848
Nos1	NM_008712	−2,136
Ntm	NM_172290	+1,156
Olfm2	NM_173777	+748
Pax7	NM_011039	+1,098
Pcp2	NM_001129804	+1,888
Pik3ap1	NM_031376	+1,893
Rcvrn	NM_009038	+7,729
Rims3	NM_182929	+4,777
Samd5	NM_177271	+2,022
St8sia6	NM_145838	+3,917
Syde1	NM_027875	+5,800
Tmem63c	NM_172583	+1,637
Tnfrsf13b	NM_021349	+1715
Ttc9	NM_001033149	−2,004
Ttyh1	NM_021324	+660
Vwa5b2	NM_001144953	+2,508

### Interaction Between miRNAs and Aire-dependent PTA mRNAs

In order to evaluate whether the increase or reduction in the PTA mRNA 3′ UTRs affected their predicted interactions with miRNAs, we used the miRDB database that initially evaluates the potential complementarity of a given miRNA seed region to the 3′ UTR of a target mRNA. Firstly, we identified the miRNAs that interact with the 3'UTR regions of PTA mRNAs of the mouse reference transcriptome that is available in the GenBank NCBI (https://www.ncbi.nlm.nih.gov/genbank/) database, and these results were considered as a reference for further analysis. We then used the 3'UTR mRNA sequences from the mTEC RNA-seq data to compare the same PTA mRNAs from this study that showed their increased or reduced 3' UTRs. This type of analysis allowed us to qualitatively and quantitatively evaluate the miRNA-mRNA interactions as well as to predict new interactions (Table 3).

**Table 3:** miRNAs that hybridize with 3'UTRs of reference PTA mRNAs or with Aire-dependent PTA mRNAs expressed by Aire KO mTECs.

**Gene symbol**	**miRNAs that interact with reference mRNAs 3'UTR**	**miRNAs that interact with alternative mRNAs 3'UTR**
Nos 1	**miR-466k, miR-7073-5p, miR-7017-5p**	**miR-7073-5p, miR-6992-5p**
Ttc9	miR-3057-3p, miR-767	*miR-3057-3p, miR-705*, miR-7008-5p
AnKrd22	miR-7093-3p	miR-7093-3p, miR-6896-5p, **miR-7685-5p**
Apobec3	**miR-219c-5p**	miR-6928-5p, *1933-3p*
Abo	miR-708-5p, miR-28a-5p	miR-708-5p, miR-28a-5p, **miR-6943-5p**
Bcl2l15	*miR-3097-3p, miR-290a-5p, miR-653-5p, miR-3112-3p, miR-185-3p, miR-543-5p*,miR-*3082-5p, miR-3092-5p*	miR-6923-3p, miR-881-5p, miR-7221-5p
Clqtnf9	miR-3090-3p, **miR-7036a-5p**, miR-124-5p	miR-3090-3p, **miR-7036a-5p, miR-6989-5p**, miR-124-5p
Caly	miR-5126	miR-92a-2-5p, **miR-383-5p,miR-7081-3p**
Chodl	miR-5098, miR-6996-3p	miR-5098, miR-6996-3p
Clcf1	miR-6372, **miR-691**	**miR-374c-5p, miR-6372**
Clcn1	miR-6989-5p	*miR-423-5p*, **miR-190a-3p**
Col23a1	miR-344d-3-5p	miR-344d-3-5p
Cxcr6	miR-466l-5p, miR-6924-5p	*miR-466l-5p*, miR-6924-5p
Dock3	miR-7231-5p, miR-7088-5p, miR-7053-5p, miR-3092-5p	miR-7231-5p, miR-7053-5p, miR-3092-5p
Fam131b	**miR-6799-5p, miR-7-5p**	**miR-3202, miR-4492**
Gfra3	**miR-6810-5p, miR-4455**	*miR-466K, miR-466d*, miR-5133, *miR-129b-3p*, **miR7048-5p**, miR-185-3p, miR-7017-5p, **miR-7094-3e**
Gjb4	miR-1264-5p	miR-466k*, miR-465d-5p*, **miR-6923-5p, miR-5110**
H2-T24	miR-7241-3p	*miR-1946a*, miR-7241-3p
Hpca	miR-466k,miR-466l-5p, miR-466d-5p	*miR-466k, miR-466d-5p*, **miR-7119-5p, miR-7024-5p**
Kcn54	miR-7052-5p	miR-7052-5p
Krt80	miR-7672-3p, miR-5113, miR-7037-5p	miR-6982-5p, miR-7672-3p, *miR-546, miR-709*
Lgi4	miR-760-3p	*miR-760-3p, miR-466n-5p*
Morn3	**miR-3074-2-3p, miR-3074-2-3p**	**miR-7076-5p, miR-7075-5p**, **miR-7030-5p**, miR-3074-3-3p
Naalad11	not found	**miR-7058-5p, miR-504-3p**
Rcvrn	miR-4763-3p, **miR-6754-5p**	**miR-7076-5p, miR-7075-5p**
Samd5	miR-7094b-2-5p, miR-5114	*miR-466k, miR-466d*
St8sia6	miR-7007-5p, **miR-3618-3p**	*miR-705*, miR-7008-5p
Syde1	miR-6975-5p, miR-504-3p	miR-6975-5p, *miR-1187*
Tmem63C	miR-6987-5p	miR-6987-5p
Ttyh1	miR-6931-5p, miR-7032-3p	miR-1249-5p, miR-6931-5p
Vwa5b2	miR-466i-5p, miR-198-5p	*miR-466i-5p*, miR-1982-5p

Increased (+) or reduced (-) 3'UTRs are indicated.

*The miRNAs highlighted in bold represent new predicted interactions (this study) and in italic are those expressed in mTECs*.

All miRNA-mRNA interactions showed in Table 3 were validated *in silico* through the RNA-Hybrid program. The results revealed that the majority of interactions show perfect complementarity between the miRNA seed region and the 3'UTR sequence of the target mRNA with mfe ≤ −20 kcal / mol. Here we have exemplified only the miRNA-mRNA interactions of the two mRNA targets that showed reduction of the 3'UTR sequence i.e., the Nos1 and Ttc9 mRNAs and of the two targets that showed increase of their 3'UTRs, i.e., Cxcr6 and Rcvrn mRNAs, respectively (Supplementary Figures 1–4).

Supplementary Figure 1 show that when we compared the interaction between the seed region of miRNA 4661-5p or miRNA 6924-5p with the 3'UTR region of the GenBank reference mRNA Cxr6 with the result obtained with mTECs (this study) for Cxcr6 mRNA, we observed that increased 3 'UTR sequence of this PTA mRNA does not unbalance the miRNA-mRNA interactions. This increase favors energetically stable interactions presenting mfe of −25.6 kcal/mol (miR-4661-5p and Cxcr6) and−26 kcal/mol (miR-6924-5p and Cxcr6).

With respect to the Rcvrn mRNA, Supplementary Figure 2 shows that the 3'UTR of this reference mRNA predicts interaction with the seed region of miRNA 4763-3p and miRNA 6754-5p, which present mfe of −35 kcal/mol and −29.3 kcal/mol, respectively. The increase of 3'UTR of mRNA Rcvrn observed in mTECs created new interactions involving miRNA 7076-5p and miRNA 7075-5p, mfe −32.7 kcal/mol and −28.9 kcal/mol, respectively.

Supplementary Figure 3 shows that the reference Nos1 mRNA interacts with the seed region of miRNA-466k, miRNA-7073-5p and miRNA-7017-5, mfe of −25.4, −25.7, and −30.2 kcal/mol, respectively. The reduction of Nos1 mRNA 3′UTR in mTECs created interaction with two new miRNAs, miRNA-7073-5p and miRNA-6992-5p, mfe −25.7, and −31.7, respectively.

Supplementary Figure 4 shows that the 3'UTR of the reference Ttc9 mRNA predicts interaction with the seed region of miRNA-3057-3p and miRNA-767, mfe of −31.5 kcal/mol and −27.7 kcal/mol, respectively. The 3'UTR reduction of the Ttc9 mRNA observed in mTECs did not alter the interaction with miRNA 3057-30, but created two new possibilities of interaction involving miRNA-705 and miRNA-7008-5p, mfe of −31.5, −37.8, and −31.3 Kcal/mol, respectively.

### PTA Self-Representation

**Figure 4:**
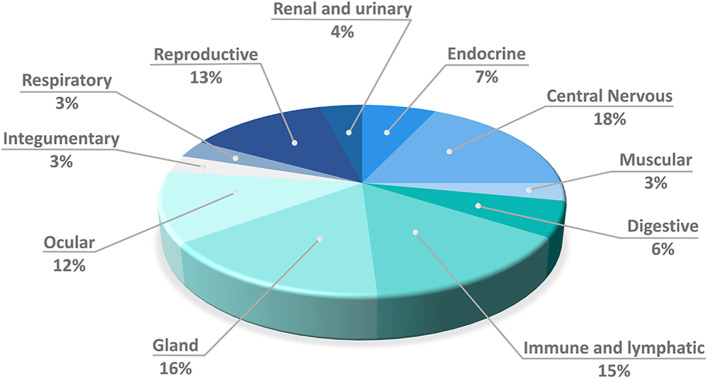
Distribution of the expressed mRNAs that encode peripheral tissue antigens into 11 anatomic/physiologic body's systems, which guarantee their self-representation through promiscuous gene expression by mTECs.

The 41 mRNAs that encode PTAs and the featured 3'UTR alterations were assigned to 11 anatomic functional body systems as follows (in descending order): central nervous, glands, immune and lymphatic, reproductive, ocular, endocrine, digestive, renal and urinary, muscular, integumentary and respiratory (Figure 4).

### Functional Enrichment of Non-PTA Aire-dependent mRNAs

**Figure 5:**
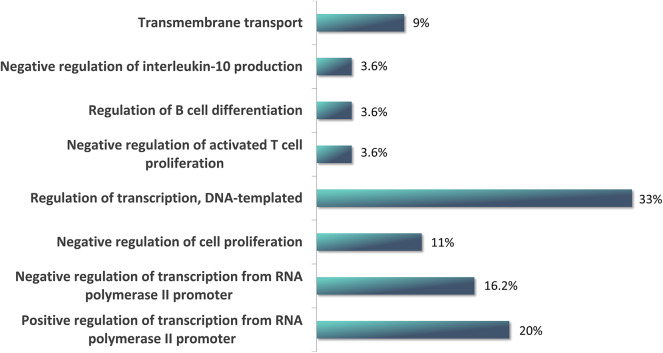
Functional enrichment of Aire-dependent non-peripheral tissue antigen (non-PTA) mRNAs, which have altered the size of the 3'UTR. Note that the most represented biological process in which these mRNAs were involved is the regulation of transcription, DNA-templated.

We performed the functional enrichment of mRNAs that do not encode PTAs, but which are Aire-dependent and which have altered the size of the 3′UTR region (Table 2). The biological process most represented by these mRNAs is relative to the regulation of transcription (Figure 5) including the transcription factors (Tbx6, Zfpm1, Crebbp, Olig2, Ikzf3, Kdm4d, Ar, Zfp605, Pura, and Zfp382).

### Identification of Alternative Polyadenylation (APA) Sites in Aire-dependent PTA-mRNAs

Table 4 shows the positioning of APA sites throughout the nucleotide sequence of the Aire-dependent PTA mRNAs. Those known APA sites for each mRNA, those most frequent and those APA sites not previously described, but which were identified in the mTEC cell samples studied, could be located. We observed that the number of APA sites does not change significantly between WT and Aire KO mTEC cells. However, we observed new APA sites in PTA mRNAs expressed by this cell type.

**Table 4:** Identification (nucleotide position) of alternative polyadenylation (APA) sites in Aire-dependent PTA mRNAs.

**mRNA symbol**	**APA described in the reference genome**	**APA found in mTECs**
Abo		26698022
Ankrd22	*34197463*, 34197421, 34197262, 34197186, 34197048, 34196687, 34196611	**34197038**
Apobec2	*48569155*, 48563164, 48558556	48558555
Bcl2l15	*103642569*, 103657600, 103657828, 103658529	103642570
Caly	147261551, *147261178*, 147261150, 147255786	**147255778**
Chodl	78951973	78951973
Clcf1		4222615
Col23a1	51162660, 51172363, 51394611, 51394879	**51394455**
Cxcr6	123720684, 123720744, *123720874*	123720871
Dock3	107131314, 107104099, 106922700, 106899728, 106880197, 106819384, 106819329, 106795438, 106795165	**106795155**
Fam131b	42265716, 42265313	**4226530**
Gfra3	*34849558*	34849556
Gjb4	127028331	127028329
H2-T24	36154872, 36151766, *36151571* 36144032, 36142991	**36142639**
Kcnj4		7931414
Krt80	101178754	**101180001**
Lgi4	31855934	**31855951**
Mcoln3	145785116, 145801541, 145802755, *145804476*	**145803609**
Nav3	109430327, 109424390, 109420674, 109413945, 109409605, 109370159, 109311523, 109307159, 109306329, 109266622, 109252998, 109240298, 109182064, 109169910, 109158838, 109137357, 109119595	**109120494**
Pax7		**139293995**
Pcp2	3623378	**3623370**
Pik3ap1	*41348601*, 41348106	**41348707**
Rims3	120556651, 120556806, 120564406, 120564591	**120564162**
Samd5	9388230, 9383240, 9381768, 9380353, 9379489, 9379423, 9376727, 9376572, 9370935, 9365563, 9364677, 9364412, 9355627, 9354377, 9352851, 9348368, 9347200, *9347058*, 9347015, 9346831, 9346649	9347056
St8sia6	13688872, 13688819, 13665851, *13577731*, 13577346, 13576980, 13576664	**13576564**
Tmem63c	88408604, 88430983	**88430987**
Ttc9	8894011, 8893567, 8893241, 8892977, 8890450, 8890170, 8889241, 8886079, 8886007, 8885939, 8885786, 8885496, 8885285, 8885175, 8885098, 8884619, 8884567, 8883673, 8883558	**82765737**
Ttyh1	4085846, 4086424, 4086530, 4086947, 4087801	**4086849**
Vwa5b2	20605449	**20605448**

*Italic, most frequent APA; bold, new APA (this study)*.

## Discussion

In this study, we analyzed two large groups of mRNAs that were expressed in medullary thymic epithelial cells (mTECs) isolated from autoimmune regulator (Aire) wild type (WT) or Aire-knockout (KO) mice (41). The first group of mRNAs encode peripheral tissue antigens (PTAs), and the other encode proteins involved with transcription control including transcription factors (TFs). The analysis was done to assess the hypothesis that Aire regulates the mRNA 3'UTR length, the preferential region where are found the binding sites of miRNAs.

In previous studies, we have observed that Aire regulates a set of miRNAs in mTECs (52), which establish miRNA-mRNA network and regulate a set of downstream Aire-dependent mRNAs (45). Interestingly, reduction in Aire in these cells imbalance miRNA-mRNA interactions and a larger set of miRNAs now act on the target mRNAs (45).

These observations raised questions about the factor (s) that lead to increased miRNA diversity acting on mRNAs (3, 44, 45). In our view, factors such as stoichiometry between miRNAs and mRNA targets or stability of base pairing may have influenced the miRNA interaction. The stoichiometry between these RNA species is expected to vary during the cell cycle and does not explain the augmentation of miRNA diversity that interact with their mRNA targets.

The Gibbs free energy or minimum free energy (mfe) ≤ −20 is considered optimal for maintaining miRNA-mRNA pairing and this value is widely used as a cutoff in the search for stable miRNA interactions. Even so, different miRNAs could still play a role at different mfe values (51). In our view, a plausible explanation for the increase in the number of miRNAs that interact with target mRNAs would be the modification in the target, most likely in the 3'UTR region. To act, these miRNAs would have to have more binding sites in the target 3'UTR region.

Variations in the structure of Aire-dependent PTA mRNAs involving the number of 3'UTR miRNA binding sites could be the most obvious cause influencing the greater variety of controller miRNAs in Aire-downregulated mTEC cells. Variations in the number of 3'UTR binding sites of Aire-dependent PTA mRNAs may represent a possible factor that influence the miRNA interaction (3, 52) an aspect that has been pursued in our group and independently by the group of M. Giraud (C. Guyon) at the INSERM (U1016) in Paris and at the University of Nantes, France (3).

As we previously asked (3), what would be the consequences of miRNA action on these PTAs? Could the different miRNAs in mTECs control autoantigen protein synthesis and consequently control the self-tolerance induction? Could changes in the length of mRNA 3′UTRs influence the action of miRNAs? Previously, we stated that there might be changes in length of the 3′UTR sequence of Aire-dependent mRNAs expressed in mTECs (3, 45, 52).

To test this hypothesis, we analyzed the length of 3′UTRs of a large set of Aire-dependent mRNAs whose data were obtained from existing RNA-seq data of mTECs isolated of wild type or Aire-knockout (KO) mice. In addition to PTAs, we also analyze the variations of the 3′UTRs of mRNAs that encode transcription factors, as these elements are at the basis of transcriptional gene expression control, which may influence self-representation by mTECs.

The use of the 3USS software allowed us to identify the existence of alternative 3'UTR sequences when comparing the mTEC wild type cell transcript set with the Aire KO mTECs. Both samples showed shortening or lengthening in this region and this demonstrated that Aire influences the generation of mRNA isoforms with respect to the 3'UTR region.

The implication of this is associated to immune homeostasis, which is largely associated to self-non-self discrimination by T-cells, a process that occurs in the thymic stroma during the development of thymocytes. These cells in population are exposed to a myriad of peripheral tissue antigen (PTA) peptides that are expressed and presented via MHC-II by medullary thymic epithelial cells (mTECs) to thymocytes (3–5, 10, 11). The greater the diversity of autoantigens presented by mTECs, the more comprehensive and at the same time accurate will be the self-tolerance of the T-cell population able to migrate from thymus to the periphery (3, 53, 54). This process must be finely controlled, if not, loss of tolerance may occur with consequences in the emergence of autoimmune reactions.

For example, our results showed that in the absence of Aire, the mRNA encoding the PTA Rcvnr, increased by 7,729 nucleotides in its 3'UTR and, consequently, the generation of two new miRNA binding sites (for miR-7076-5p and miR−7075-5p). As this PTA represents retinal pigment epithelium, retina and iris, which are target during autoimmune reactivity, this suggests a mechanism of autoimmunity of the eye structures. Similarly, the mRNA that encodes the PTA Syde-1 had an increase of 5,800 nucleotides in its 3'UTR with the appearance of a new miR-1187 binding site. This PTA represents ovary and adrenal glands that are target organs for autoimmune reactivity in APECED syndrome. This illustrates in what way changes in the 3'UTR of mRNAs could clinically affect aggressive autoimmunity.

Two other mRNAs that encode proteins involved in the biology of mTECs, such as Crebbp (an AIRE partner) and Ago-2 (a key element in the RISC complex during the miRNA mechanism of action), also had an increase in their 3′UTRs and consequently the generation of new miRNA binding sites. CREBBP being negatively regulated by miRNAs may destabilize the AIRE complex in the chromatin of mTECs with consequences for PGE control. In addition, Ago-2 being negatively regulated might cause a general imbalance in the posttranscriptional control.

Aire gene/protein is the main transcriptional regulator of PTAs that acts in synergy with Forebrain embryonic zinc finger-like protein 2 (FEZF2) in mTECs (4–9). Although Aire is an upstream transcriptional controller, this gene / protein has a link to posttranscriptional control in mTECs (11, 45, 52). In addition to evaluating variations in the length of 3'UTR sequences of the Aire-dependent mRNAs, we also examined whether these variations could affect miRNA-mRNA interactions.

Initially, it was necessary to establish a reference for comparing miRNA interactions with known target PTA mRNAs by making use of miRDB database. This approach enable us to compare and to evaluate the effect of Aire on both the length of mRNA 3'UTRs and the number of different miRNAs they interact, i.e., we treated the data qualitatively and quantitatively. We use as a criterion for thermodynamic stability of the interactions between the seed regions of the miRNAs and the 3'UTR of the target mRNAs the score of minimal free energy (mfe ≤ −20). This criterion allowed the development of a thermodynamically favorable miRNA-mRNA interaction prediction algorithm (51), which is a widely used method for different model-systems.

We show that Aire interferes with the 3'UTR length of Aire-dependent mRNAs and that its absence modify the composition of miRNA-mRNA interactions creating new miRNA sites on PTA mRNAs. This might have influence in reduced PTA protein translation. We evidence that absence of Aire not only interferes directly with transcription of PTAs, but also with the structure of its respective mRNAs.

As previously shown (41, 43, 55) Aire also controls the expression of non-PTA mRNAs. In this study, it was found that those mRNAs associated with transcription control, including those encoding transcription factors, were also under Aire control that affected the size of their respective 3'UTRs. This certainly leads to an imbalance in the overall gene expression of mTECs with implications for self-representation. Although FEZF2 plays its role as a TF in mTECs that works in synergy to AIRE, we were not able to evidentiate its transcriptional expression in the samples analyzed.

It is well-known that the maturation of the 3′ ends of most eukaryotic mRNAs is dependent on cleavage and polyadenylation (C/P) and that this RNA species has several C/P sites. Processing of mRNAs by C/P, which involves endonucleolytic cleavage of the nascent RNA and then addition of the poly (A) tail is one of the processes of control of gene expression in eukaryotes (56–60).

The site where polyadenylation occurs, the poly(A) site, is so important that mutations at this region are associated with human diseases such as thrombophilia to some forms of thalassemias and cancer as well (57, 59, 61, 62). In addition, shortening or lengthening and polymorphisms of 3'untranslated region (3'UTR) corresponds to a source of mRNA isoforms and this process is associated to some human diseases including autoimmune diseases (60, 63–65).

However, under physiological conditions eukaryotic genes express isoforms that show differences in the 3' end of their respective mRNAs. The mRNAs from a given gene but differing at their 3' end are translated into proteins that show structural and functional differences. In addition, 3' end processing may involve the half-life of the transcribed mRNA due to inclusion or exclusion of regulatory elements, as a rule miRNA binding sites. The absence or presence of these elements implies the biological properties of mRNA/protein such as export, abundance, stability, location, and transport and translation rate (59, 62).

AIRE protein is also involved with these processes either interacting with proteins of pre-mRNA processing (1), alternative splicing (38) and polyadenylation factors (39). Then, we investigated whether Aire could affect the number of APA sites of Aire-dependent mRNAs. Our results show that rather reflecting the presence or absence of Aire, the new APA sites in mRNAs is more associated to mTEC cell type. This is still an open question to search for genes/proteins involved in this process.

Overall, these results point toward the existence of a controlling mechanism in mTECs in which Aire gene/protein affects the length of 3'UTR of Aire-dependent mRNAs. Under absence of Aire, the mRNA targets feature increased sequence were is found miRNA binding sites. The miRNAs then exert posttranscriptional control over these mRNAs and might decrease protein translation that in the case of PTAs influence self-representation in the thymus.

## Data Availability Statement

The datasets generated for this study can be found in the GEO acc number GSE65617.

## Author Contributions

EO: conceived the study and performed data analysis. AA: performed data analysis. CS-H: performed data analysis. MD: performed data analysis. GP: conceived the study, chose the analysis tools, and wrote the manuscript.

## Conflict of Interest

The authors declare that the research was conducted in the absence of any commercial or financial relationships that could be construed as a potential conflict of interest. The reviewer PP declared a past co-authorship with one of the authors GP to the handling editor.

## Supplementary Material

Supplemental Figure 1 Molecular interactions between miRNAs and mRNA targets that feature reduction or increase in their 3'UTRs and their respective thermodynamic minimal free energy (mfe). Comparison of the interaction between the seed region of miRNA 4661-5p or miRNA 6924-5p with the reference 3'UTR (GenBank NCBI) of mRNA Cxr6 with the respective interaction obtained with mTECs (this study) for Cxcr6 mRNA.

Supplemental Figure 2 Molecular interactions between miRNAs and mRNA targets that feature reduction or increase in their 3'UTRs and their respective thermodynamic minimal free energy (mfe). The increase of 3'UTR of mRNA Rcvrn observed in mTECs created new interactions for miRNA 7076-5p and miRNA 7075-5p.

Supplemental Figure 3 Molecular interactions between miRNAs and mRNA targets that feature reduction or increase in their 3'UTRs and their respective thermodynamic minimal free energy (mfe). The reduction of Nos1 mRNA 3'UTR in mTECs created interaction with two new miRNAs, miRNA-7073-5p and miRNA-6992-5p.

Supplemental Figure 4 Molecular interactions between miRNAs and mRNA targets that feature reduction or increase in their 3'UTRs and their respective thermodynamic minimal free energy (mfe). The 3'UTR reduction of the Ttc9 mRNA observed in mTECs did not alter the interaction with miRNA 3057-30, but created two new possibilities of interaction involving miRNA-705 and miRNA-7008-5p.
