# Supplementary figures and images for: Aire Gene Influences the Length of the 3′ UTR of mRNAs in Medullary Thymic Epithelial Cells
Source: Front Immunol. 2020 May 28;11:1039. doi: 10.3389/fimmu.2020.01039 (PMC7270294; doi:10.3389/fimmu.2020.01039)

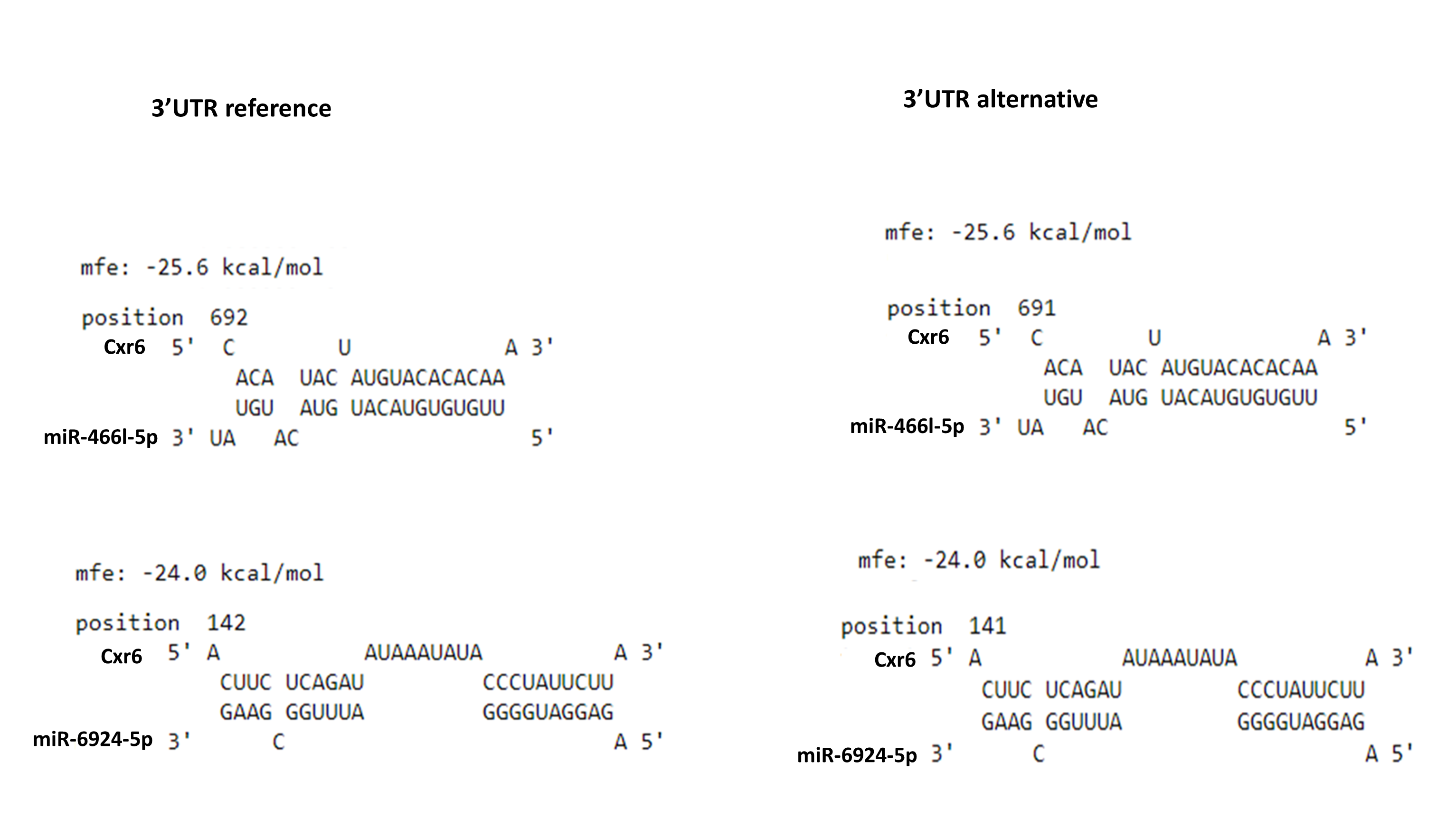

Supplement: Supplemental Figure 1 — Molecular interactions between miRNAs and mRNA targets that feature reduction or increase in their 3'UTRs and their respective thermodynamic minimal free energy (mfe). Comparison of the interaction between the seed region of miRNA 4661-5p or miRNA 6924-5p with the reference 3'UTR (GenBank NCBI) of mRNA Cxr6 with the respective interaction obtained with mTECs (this study) for Cxcr6 mRNA. [file Image_1.TIF]

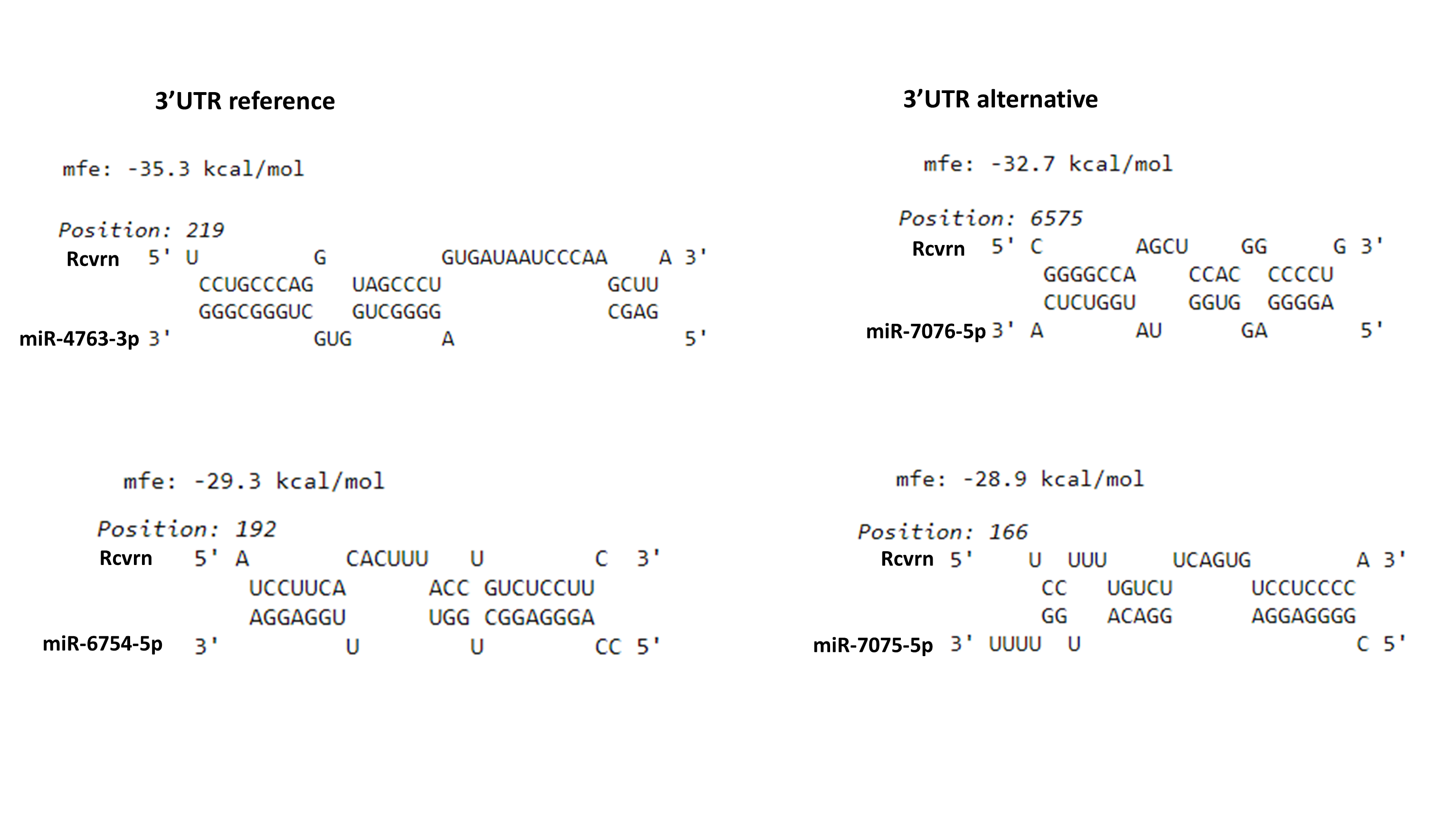

Supplement: Supplemental Figure 2 — Molecular interactions between miRNAs and mRNA targets that feature reduction or increase in their 3'UTRs and their respective thermodynamic minimal free energy (mfe). The increase of 3'UTR of mRNA Rcvrn observed in mTECs created new interactions for miRNA 7076-5p and miRNA 7075-5p. [file Image_2.TIF]

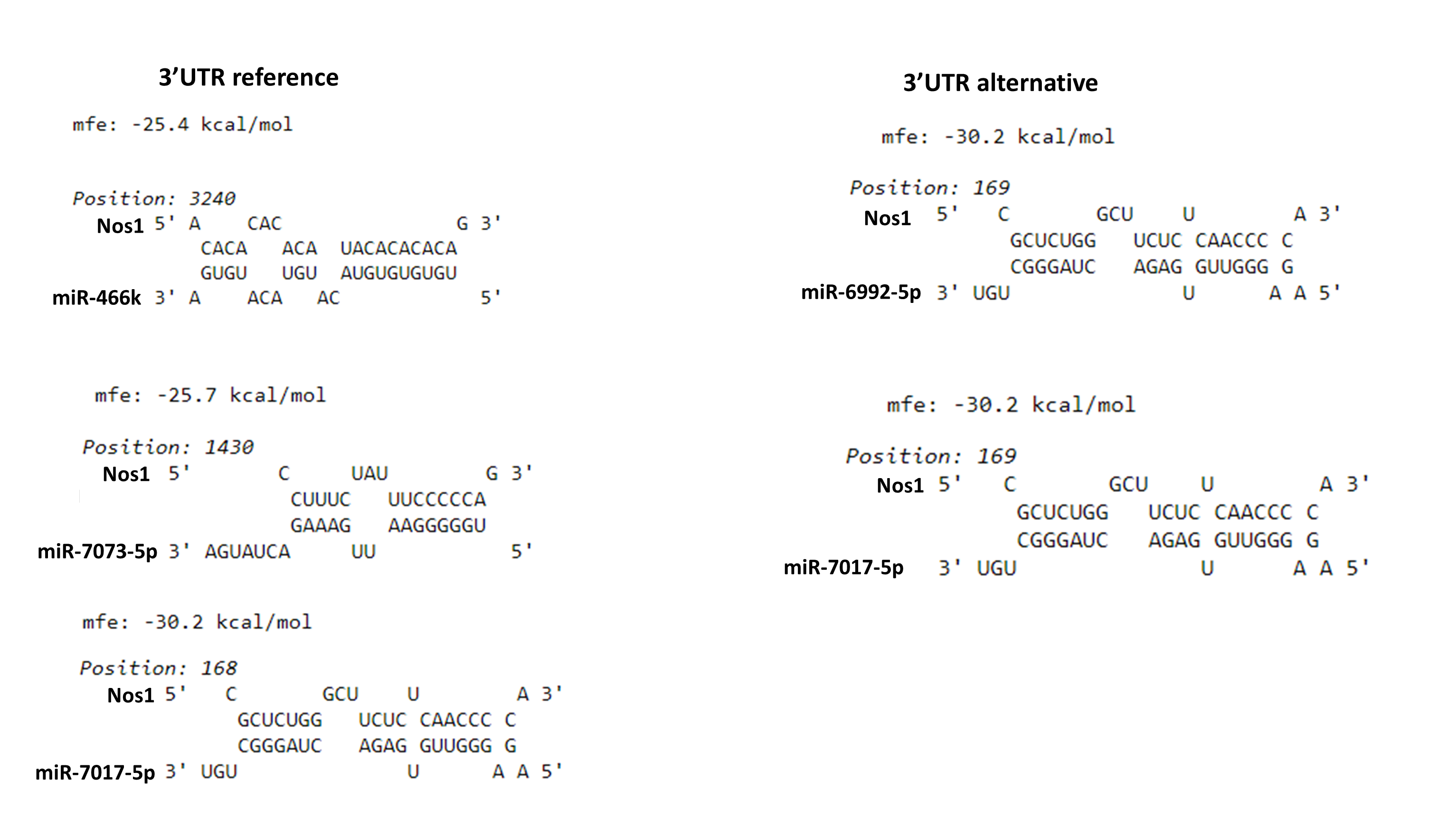

Supplement: Supplemental Figure 3 — Molecular interactions between miRNAs and mRNA targets that feature reduction or increase in their 3'UTRs and their respective thermodynamic minimal free energy (mfe). The reduction of Nos1 mRNA 3'UTR in mTECs created interaction with two new miRNAs, miRNA-7073-5p and miRNA-6992-5p. [file Image_3.TIF]

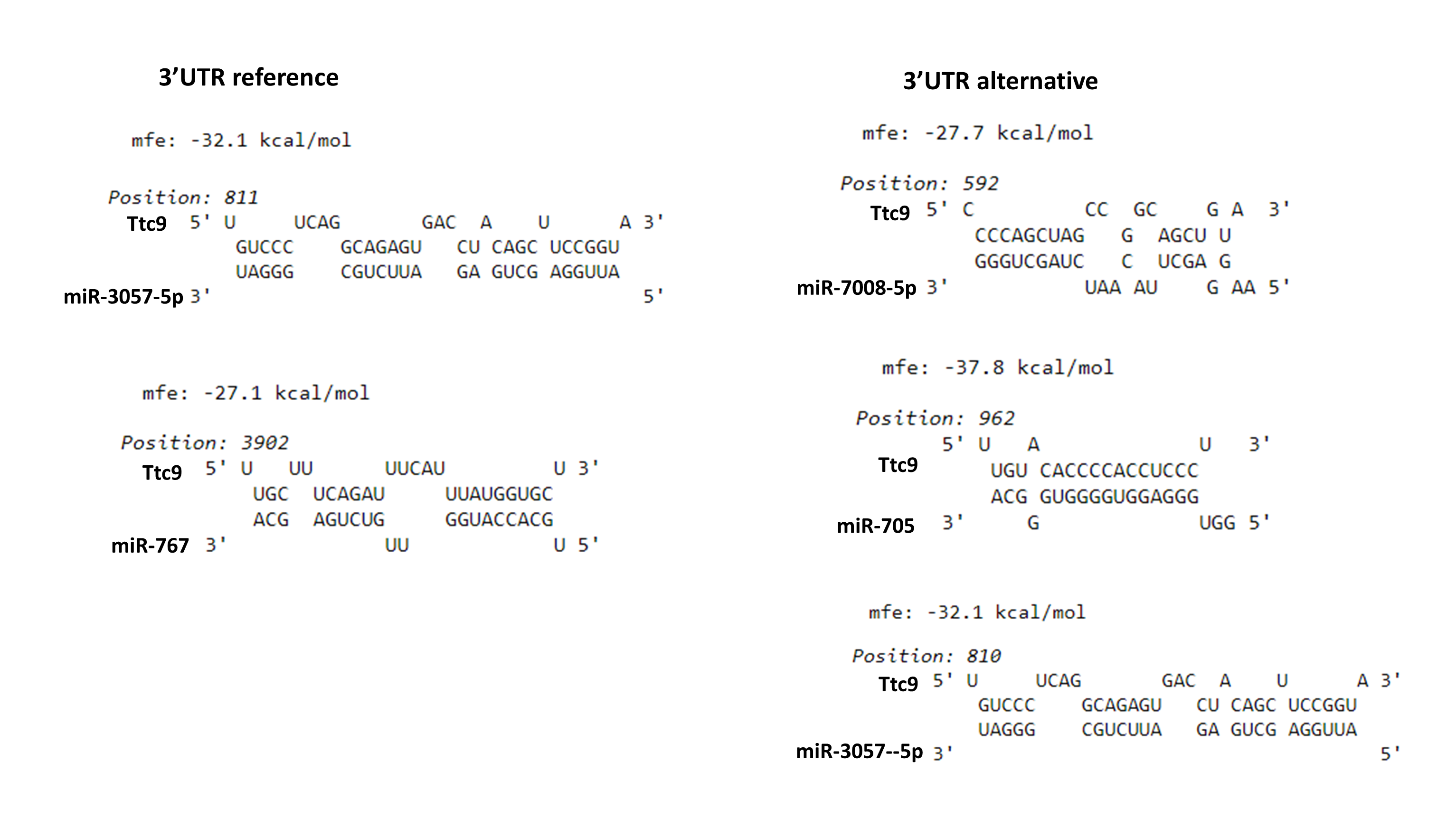

Supplement: Supplemental Figure 4 — Molecular interactions between miRNAs and mRNA targets that feature reduction or increase in their 3'UTRs and their respective thermodynamic minimal free energy (mfe). The 3'UTR reduction of the Ttc9 mRNA observed in mTECs did not alter the interaction with miRNA 3057-30, but created two new possibilities of interaction involving miRNA-705 and miRNA-7008-5p. [file Image_4.TIF]
