# Supplementary material for: Aire Gene Influences the Length of the 3′ UTR of mRNAs in Medullary Thymic Epithelial Cells
Source: Front Immunol. 2020 May 28;11:1039. doi: 10.3389/fimmu.2020.01039 (PMC7270294; doi:10.3389/fimmu.2020.01039)
Supplement: Supplementary file 5 [file Table_1.docx]

**Supp Table 1.** Differentially expressed PTA and non-PTA mRNAs and their respective log fold-change, log CPM, P value and FDR used to construct the hierarchical clustering.

| Gene symbol | logFC | logCPM | PValue | FDR |
| --- | --- | --- | --- | --- |
| 1700008O03Rik | 0.709923881140732 | 0.705266432303783 | 0.00134333166019613 | 0.00276239909559653 |
| 4930503L19Rik | -1.47608992634608 | 0.72362206842441 | 6.06408672023205e-09 | 2.1289947525683e-08 |
| 9130011E15Rik | 0.624183072785589 | 3.86483497544509 | 5.79392581939546e-09 | 2.03832042898697e-08 |
| Abo | -2.63666354283852 | -0.184613993214077 | 6.50533729222123e-14 | 3.24388312930209e-13 |
| Acpt | -3.60379209810943 | 0.329211796987145 | 1.61654865462565e-30 | 1.79118964501457e-29 |
| Acvr2b | -0.799213953131913 | 0.0594636950565884 | 0.00232819737903609 | 0.00462249645076656 |
| Adora2a | 0.728215084271262 | 5.1307913795533 | 9.50366870148785e-16 | 5.26276070225446e-15 |
| Ago-2 | 0.592399332091652 | 5.97515909028662 | 2.46336948036143e-11 | 1.03249855970441e-10 |
| Ankle1 | -0.838703349555179 | 1.54797114640805 | 4.64054301772224e-06 | 1.26993195467883e-05 |
| Ankrd13d | -0.80160146115923 | 1.64977620076183 | 2.09756833776165e-06 | 5.93511437235081e-06 |
| Ankrd22 | -1.16307928283664 | 3.08729553520852 | 2.00324915296256e-18 | 1.28653946136209e-17 |
| Ap5b1 | 0.916851271109192 | 4.64737655838559 | 6.17732930371326e-21 | 4.50078637793316e-20 |
| Apobec2 | -2.8104663628643 | 0.285375298409324 | 1.01306758351182e-18 | 6.62316044791164e-18 |
| Ar | 0.99130662536623 | 3.28027067549625 | 1.58903077691974e-16 | 9.1984415451215e-16 |
| AY074887 | 1.3016373891334 | -1.88821807339808 | 0.0165433243931722 | 0.0287480838053822 |
| Bcl2l15 | -1.35519027210244 | 4.33257769717124 | 4.09229687395328e-50 | 9.00230293078631e-49 |
| Bora | -0.844074032731263 | 2.38354758754629 | 3.8688252613157e-08 | 1.27598250285515e-07 |
| Btn2a2 | 1.22020698802197 | 3.08029415174147 | 5.61023553924065e-18 | 3.53493444320755e-17 |
| C1qtnf9 | -2.16328314475447 | 0.15816900403191 | 7.81410516978886e-15 | 4.11720140669446e-14 |
| Cacna1e | 1.05254079869108 | 5.03883270668607 | 1.33157801637603e-22 | 1.05124580240213e-21 |
| Calml3 | 0.65304757412188 | 6.09075093425744 | 3.25208064752041e-16 | 1.85215793878713e-15 |
| Caly | -1.95103015797732 | 0.676997207424027 | 4.64050774442096e-16 | 2.61867354493541e-15 |
| Camkv | -0.749658227222994 | 2.84303398316797 | 1.39343624496291e-09 | 5.15689589500538e-09 |
| Car13 | -1.26641532025887 | 3.21086356839951 | 2.62501946263877e-29 | 2.77168794999255e-28 |
| Cbx7 | 0.790060048194339 | 6.32829951260039 | 2.00269253713314e-26 | 1.85792890959394e-25 |
| Ccdc78 | -1.35107963633073 | 0.748310106929652 | 2.22004876396677e-06 | 6.26763561171656e-06 |
| Ccdc87 | 0.785971412023766 | 3.83784429138588 | 3.59454841191124e-15 | 1.93212008702956e-14 |
| Cd79a | -1.51068696273142 | 0.766695043453812 | 2.51983875130729e-08 | 8.425206189938e-08 |
| Cdh13 | -1.4519843665158 | 0.809210561290801 | 1.02624848184014e-09 | 3.83584543905361e-09 |
| Cdh8 | 2.02236370083663 | 5.68895624162224 | 1.58579195586895e-162 | 3.65951989815912e-160 |
| Chodl | -4.53496961626237 | 0.827100682032016 | 2.96323796476952e-34 | 3.75489499231618e-33 |
| Chrna2 | -1.18330862413177 | 2.56468478803134 | 8.24533836179897e-14 | 4.0801674367665e-13 |
| Clcf1 | -1.49304040701756 | 0.987667699729781 | 3.28033838014296e-08 | 1.08770545901397e-07 |
| Clcn1 | -1.12454134263358 | 2.08668251106499 | 4.95557745374154e-05 | 0.000121870948754787 |
| Cldn20 | -1.02311695502251 | -0.244080556004447 | 0.00120144313386012 | 0.0024874599044723 |
| Clmp | 1.06188788244723 | 4.6344560554217 | 9.92370306586896e-37 | 1.40264354287901e-35 |
| Col23a1 | -3.71530517454583 | 1.70861128858816 | 6.03449664648917e-56 | 1.57421651647543e-54 |
| Coro6 | -2.03639838058744 | 1.12779096499761 | 3.81087748755276e-19 | 2.5427039116282e-18 |
| Crebbp | 1.42642889781039 | 6.42811838616877 | 1.26736260990484e-47 | 2.6108757628941e-46 |
| Ctf2 | -1.47106837855276 | -1.84380890527211 | 0.00450847377706621 | 0.00858484375195089 |
| Cxcr6 | -1.72481551156042 | 0.194556901154926 | 4.46245194916115e-10 | 1.71880672090945e-09 |
| Cyp8b1 | 1.36062396053649 | 1.51326852765026 | 1.43067149998283e-14 | 7.41600777528898e-14 |
| Cyyr1 | 1.59509283597958 | 3.70682299699102 | 1.5865827792284e-37 | 2.31617923974948e-36 |
| Degs2 | -0.907454756275376 | 2.51737022485554 | 1.27528066452722e-11 | 5.4625621896579e-11 |
| Dgki | -0.806346480727527 | 2.76346894554628 | 6.73978308169496e-07 | 1.99894703362184e-06 |
| Dock3 | -1.78867723015517 | 2.50057196604815 | 5.58108610797423e-28 | 5.53496142939593e-27 |
| Dstyk | -0.650529031182686 | 4.0716000785907 | 3.37159296091692e-10 | 1.31062894496284e-09 |
| Dusp4 | 0.760305923902012 | 7.79048089530132 | 3.65519599792117e-28 | 3.64911414101947e-27 |
| Eng | -1.30220513295779 | 1.48306686140101 | 8.21998191644026e-05 | 0.000196709109576794 |
| Fam126a | 0.603461551056152 | 5.03461016827814 | 4.62242526492901e-15 | 2.46913435028481e-14 |
| Fam131b | -1.80295021729542 | 0.364430379099552 | 3.4907861889774e-08 | 1.15525191030692e-07 |
| Fam217b | 0.678935900433543 | 1.72936702641468 | 5.05655783846347e-05 | 0.000124277919224909 |
| Fam71e2 | 0.617498818792263 | 0.666109165011997 | 0.00499911925733067 | 0.00945832575293151 |
| Fcgr2b | -1.69979294363242 | 0.897978685231013 | 2.04185666946811e-13 | 9.8284316219885e-13 |
| Frrs1l | -2.98404755868204 | -0.967450116563538 | 1.07019405545875e-10 | 4.31095289207955e-10 |
| Gabbr2 | -2.02006103968833 | 1.6952852349159 | 4.14889667764677e-20 | 2.91065537163176e-19 |
| Gcnt7 | -1.286067485026 | 0.0847838617279231 | 0.000179718666120694 | 0.000413939346151311 |
| Gfra3 | -1.62282651824746 | 3.34032868495856 | 6.78971837433101e-37 | 9.66507953641424e-36 |
| Gjb4 | -2.28445607153829 | 0.325438446181723 | 3.40414251224686e-10 | 1.32306753512428e-09 |
| Gm4858 | -2.99822874233355 | 1.36233665640561 | 2.40112567098702e-39 | 3.76647164076396e-38 |
| Gm5617 | 0.686779392601046 | 1.67126319994536 | 0.00150669195343312 | 0.00307409732911629 |
| Gpr135 | -0.895420055435659 | -2.22637382313475 | 0.0198494491273766 | 0.272813411440292 |
| Gpr137c | -1.05720424896016 | -0.495607106821234 | 0.00074386524190974 | 0.00158213096471409 |
| Gpr171 | 0.681451363663479 | 1.44623159169206 | 0.000311464862437364 | 0.000694781736081116 |
| H2-T24 | -1.49978696485735 | 1.70657472747006 | 4.71149823272382e-18 | 2.97646637497688e-17 |
| Hpca | -2.80812685566294 | 2.30159928931678 | 7.54574767732701e-48 | 1.56523720186559e-46 |
| Hsd3b7 | 0.784786741917387 | 5.30793825703022 | 7.03560157219114e-19 | 4.64140840386442e-18 |
| Ikzf3 | -0.709890456665504 | 2.9231635383476 | 5.65848596670236e-07 | 1.68973078512948e-06 |
| Il15 | 1.66420607038494 | 6.05338394440001 | 6.68270047720173e-125 | 8.44130586593903e-123 |
| Il1a | -1.13922383708233 | 1.60412358804763 | 3.26710084712969e-07 | 9.95687877220477e-07 |
| Impg1 | 1.35327502491155 | -0.173607362359386 | 0.000352365814351517 | 0.000780505726297777 |
| Inpp5f | -1.10403666939492 | 3.29024803971908 | 1.09000558517689e-18 | 7.11453196743144e-18 |
| Jag2 | -0.737890774865435 | 4.00631935744466 | 2.81289913328176e-14 | 1.43332439912446e-13 |
| Kcng3 | 2.00430872262465 | 2.95338194822525 | 2.79181645432806e-38 | 4.20085234507044e-37 |
| Kcnj4 | -1.96437671077284 | 0.72086969739382 | 1.053633004175e-12 | 4.84243433554195e-12 |
| Kctd4 | -0.623028473610176 | 2.15185182016816 | 0.00134026607952413 | 0.00275671334887535 |
| Kdm4d | 1.69511988465094 | 2.13626941798078 | 3.16646957252923e-28 | 3.16911049794419e-27 |
| Kif13b | 0.687170420515146 | 6.43528625736075 | 7.40773607141658e-19 | 4.88019944315119e-18 |
| Krr1 | 0.869610085945262 | 4.49180891879591 | 5.51665370946491e-20 | 3.84770964914728e-19 |
| Krt80 | -1.67846969693385 | 3.92269787802455 | 6.80910499384636e-42 | 1.16644196896726e-40 |
| Lgi4 | -2.48344049430342 | 0.289370557795493 | 9.48926694315291e-14 | 4.67643545453121e-13 |
| Lman1l | -2.96687267718049 | 0.735116013936949 | 5.03358367094419e-17 | 2.99915610979793e-16 |
| Lnpep | 0.737472917766346 | 5.9720588811698 | 3.51101812026213e-15 | 1.8880671047791e-14 |
| Lrp1b | 1.61727112310197 | 4.93993896390171 | 6.88151096459729e-60 | 2.01655998962558e-58 |
| Lrrc75a | -1.0188275817493 | 0.196381679408434 | 0.000241661976696921 | 0.000548400854834162 |
| Mcoln3 | -1.69894252343076 | 0.148728607967468 | 8.96269849408983e-09 | 3.10351700848587e-08 |
| Mettl1 | -0.693806881446363 | 1.89021907190261 | 0.000265737771196155 | 0.000598958161974805 |
| Mettl11b | 0.946401279438776 | 2.61765516481611 | 1.18392848701014e-05 | 3.11594294201595e-05 |
| Mfsd9 | 1.34079335548705 | 3.75524031629569 | 1.26929330423247e-31 | 1.46456919719132e-30 |
| Mmp20 | -1.8335708357526 | 1.82376814955415 | 4.21840352210208e-18 | 2.66987564690005e-17 |
| Morn3 | -2.51710473518864 | -1.63651067625974 | 9.61639414545936e-06 | 2.55726824920803e-05 |
| Msi1 | 1.44602345444673 | 6.89446348620606 | 1.93252395068341e-54 | 4.83130987670854e-53 |
| Myo7a | -0.598855701145493 | 4.83728485118384 | 5.05595676917813e-10 | 1.93931536615431e-09 |
| Naaladl1 | -3.39031465985294 | 0.620561769030377 | 6.00545412835119e-32 | 7.01366905500869e-31 |
| Nav3 | -1.57379682452712 | 2.43456048070839 | 2.78918313562124e-17 | 1.69469355075721e-16 |
| Nos1 | -3.1080261659715 | 1.17876878289592 | 4.21945515653221e-23 | 3.40736620985104e-22 |
| Npsr1 | 1.93799043420693 | 5.14770769593497 | 2.58503920466148e-102 | 2.01431626337258e-100 |
| Ntm | -1.32163764463771 | 1.3513781204629 | 9.00269551864374e-11 | 3.64051714317523e-10 |
| Olfm2 | -1.9697307120637 | 1.33730899919201 | 1.03890635414346e-16 | 6.08288667954212e-16 |
| Olfr1137 | 1.86787986572498 | -1.8721064366948 | 0.000317592878751443 | 0.000707662156720326 |
| Olfr1138 | 3.79075284999334 | -0.0867300020989713 | 8.41079052170118e-28 | 8.29330207562976e-27 |
| Olig2 | 2.05626741279257 | 2.59549394863175 | 1.05970485135289e-48 | 2.25070056924508e-47 |
| Otud6a | 2.26308141580379 | -2.73099386384469 | 0.0282200576565334 | 0.0470073142529707 |
| Papln | -1.67656166489316 | 1.6669949193801 | 5.53762030206064e-19 | 3.66831044022786e-18 |
| Pax7 | -3.13486613931574 | 1.75133091207833 | 3.92175982341847e-57 | 1.0635280877067e-55 |
| Pcp2 | -1.89404294841651 | -1.01235013351069 | 3.24410495436882e-05 | 8.13653661875345e-05 |
| Pdcd1lg2 | 0.96631716164841 | 5.54139812747613 | 1.19008644980546e-41 | 2.01994871254109e-40 |
| Pgp | 0.823801576985285 | 3.80692874819769 | 7.8151155512901e-14 | 3.87286337458109e-13 |
| Pgpep1l | -1.12211618808614 | -0.180770524194733 | 0.000139541465469053 | 0.000325619365217041 |
| Pik3ap1 | -1.03818060298845 | 2.17920667786848 | 5.86710683543527e-08 | 1.90567822507033e-07 |
| Pou6f2 | 1.06068215826478 | 0.567897068189305 | 2.9112755866487e-05 | 7.3408924227326e-05 |
| Prorsd1 | 0.87164876270614 | 3.29717565823449 | 3.13865274454884e-13 | 1.49489315080715e-12 |
| Psg25 | -1.81172853233737 | 2.25811345224328 | 1.15475706052605e-29 | 1.24000758177294e-28 |
| Pura | 0.603335330711316 | 3.35885527483453 | 9.74144621407256e-07 | 2.84941753976528e-06 |
| Rcvrn | -2.67403877059377 | 1.43542125015733 | 5.48541535505044e-21 | 4.00273543694772e-20 |
| Rims3 | -1.56356746388916 | -0.31472606050875 | 1.52636422122863e-07 | 4.78421592131214e-07 |
| Rnf125 | 0.867463366543787 | 3.87280754937433 | 1.51223305933642e-20 | 1.08113176717528e-19 |
| S100a7a | -3.17793470897958 | 1.0349062448729 | 2.31172103808459e-28 | 2.32723594438046e-27 |
| Samd5 | -1.39356954315672 | 0.690006907006407 | 7.21008208321623e-09 | 2.51404867059697e-08 |
| Sdr9c7 | -0.602141130537928 | 4.24263898997104 | 3.43722565308463e-11 | 1.42598817068334e-10 |
| Slc46a3 | -1.05624182609066 | 2.22790497463811 | 1.93225603908697e-13 | 9.3176903632886e-13 |
| Slc9b1 | -1.40863781712869 | -1.23706705532623 | 0.0131169867821406 | 0.0232364690560508 |
| Spata17 | -0.721464186515117 | 0.00689785005135647 | 0.014691454306563 | 0.025787676688182 |
| Spata25 | -1.88399222622866 | -2.43639338218505 | 0.0200844495560972 | 0.0343617614304486 |
| Spidr | 0.868830432674121 | 4.35393130338451 | 1.6826076466723e-20 | 1.20007677622987e-19 |
| St8sia6 | -1.61257267673491 | 2.18565867876289 | 1.58806552821772e-22 | 1.24839740180888e-21 |
| Susd3 | -1.93415391657442 | -0.546452741828489 | 6.20522290872123e-09 | 2.1769529280706e-08 |
| Syde1 | -1.27643746621147 | -0.39137470476593 | 0.000180394141886308 | 0.000415415410216024 |
| Sync | 0.598723004987984 | 3.05307435954725 | 8.01673120899972e-07 | 2.36307478526152e-06 |
| Sypl2 | -0.746458685168144 | 2.39303917937326 | 2.86016090312257e-06 | 7.98277261018045e-06 |
| Syt17 | 0.985849657549994 | 2.8071907056493 | 9.52406787257776e-14 | 4.69262223243413e-13 |
| Tbl1x | 0.703143962716216 | 5.24971750677952 | 9.70539116414904e-18 | 6.03913372931234e-17 |
| Tbx6 | -0.794659696357999 | -0.580196300609465 | 0.015483803070859 | 0.0270735300670709 |
| Tcte1 | 0.637581344828939 | 3.42132035474512 | 5.2206247176857e-06 | 1.42170649295878e-05 |
| Tex101 | -1.6627404079535 | -0.465624027677925 | 1.28916373959367e-06 | 3.72232071105005e-06 |
| Tmem200b | 0.62585331138649 | 3.45653992033609 | 7.6452569774961e-08 | 2.45894086652246e-07 |
| Tmem63c | -1.72957396734826 | 3.03378125780643 | 5.89144094161789e-35 | 7.75189597581301e-34 |
| Tmod4 | 1.14716806178304 | 0.650821841079172 | 1.7321682919637e-06 | 4.9425798367767e-06 |
| Tnfrsf13b | -1.80728574855784 | -1.25306688589737 | 2.05052131276917e-05 | 5.25804869197323e-05 |
| Tnfrsf25 | -1.51266451307661 | -0.212499648163084 | 1.80542348184389e-05 | 4.65615340256322e-05 |
| Trhr2 | 0.692260200210337 | 1.87715119600605 | 0.000257597317689229 | 0.000581757375039192 |
| Ttc9 | -1.32061562383133 | 0.582073144088254 | 1.29126309523118e-05 | 3.38026988280414e-05 |
| Ttyh1 | -2.74531509472581 | 1.05949721132789 | 5.88008796878201e-24 | 4.92572814138807e-23 |
| Usp50 | -1.5294121133301 | -0.637741332399915 | 0.000639786735647179 | 0.00137330128392204 |
| Vwa5b1 | -2.53535580408794 | 1.96888687968779 | 4.65026557336672e-22 | 3.57712736412825e-21 |
| Vwa5b2 | -2.38595983513677 | -0.520770685284768 | 3.3460375457064e-07 | 1.01883914104229e-06 |
| Zbtb8os | 0.649997721363947 | 2.94650970881592 | 6.04207360864242e-05 | 0.000146964393034781 |
| Zc2hc1a | 0.843751443701684 | 5.6912451892963 | 1.95564615671888e-34 | 2.50189273780667e-33 |
| Zfp128 | 1.89156526762494 | 1.0167626534975 | 2.92481440930069e-12 | 1.3018461762466e-11 |
| Zfp382 | -0.971582594731506 | 0.887467584588728 | 1.23323921126694e-05 | 3.2364943762063e-05 |
| Zfp605 | 0.820780064598754 | 3.16244513539738 | 8.77673700392355e-13 | 4.06408813610197e-12 |
| Zfpm1 | 0.654747117879425 | 4.41812535361385 | 4.40695486795517e-14 | 2.21966247284206e-13 |
