# Supplementary material for: Aire Gene Influences the Length of the 3′ UTR of mRNAs in Medullary Thymic Epithelial Cells
Source: Front Immunol. 2020 May 28;11:1039. doi: 10.3389/fimmu.2020.01039 (PMC7270294; doi:10.3389/fimmu.2020.01039)
Supplement: Supplementary file 6 [file Table_2.docx]

| **Gene symbol** | **Official full name** | **Chromosomal location** | **Biological processes** |
| --- | --- | --- | --- |
| 1700008O03Rik | RIKEN cDNA 1700008O03 | 7 B3  NC_000073.6 | putative uncharacterized protein C19orf81 homolog |
| 4930503L19Rik | RIKEN cDNA 4930503L19 | 18 E2  **NC_000084.6** | [negative regulation of cell proliferation](http://amigo.geneontology.org/amigo/term/GO:0008285) |
| 9130011E15Rik/  Armh3 | armadillo-like helical domain containing 3 | 19 C3  NC_000085.6 | No biological process available |
| Abo | alpha 1-3-N-acetylgalactosaminyltransferase | 2 A3  NC_000068.7 | carbohydrate metabolic process, protein glycosylation, glycolipid biosynthetic process, lipid glycosylation, |
| Acpt | acid phosphatase 4 | 7 B3  NC_000073.6 | acid phosphatase activity  hydrolase activity  protein tyrosine phosphatase activity  receptor tyrosine |
| Acvr2b | activin receptor IIB | 9 F3  NC_000075.6 | negative regulation of transcription from RNA polymerase II promoter, skeletal system development, gastrulation with mouth forming second, kidney development, lymphangiogenesis, blood vessel remodeling, regulation of transcription, DNA-templated, protein phosphorylation, signal transduction, transmembrane receptor protein serine/threonine kinase signaling pathway, determination of left/right symmetry, pattern specification process, mesoderm development, heart development, response to glucose, post-embryonic development, anterior/posterior pattern specification, regulation of signal transduction, phosphorylation, insulin secretion, lung development, positive regulation of bone mineralization, BMP signaling pathway, pancreas development, activation of protein kinase activity, activin receptor signaling pathway, positive regulation of activin receptor signaling pathway, organ growth, odontogenesis of dentin-containing tooth, positive regulation of osteoblast differentiation, embryonic foregut morphogenesis, skeletal system morphogenesis, palate development, lymphatic endothelial cell differentiation, artery development, venous blood vessel development, retina vasculature development in camera-type eye, |
| Adora2a | adenosine A2a receptor | 10 C1  NC_000076.6 | synaptic transmission, dopaminergic, adenosine receptor signaling pathway, response to amphetamine, regulation of transcription, DNA-templated, negative regulation of protein kinase activity, signal transduction, G-protein coupled receptor signaling pathway, adenylate cyclase-activating G-protein coupled receptor signaling pathway, protein kinase C-activating G-protein coupled receptor signaling pathway, synaptic transmission, cholinergic, locomotory behavior, negative regulation of cell proliferation, positive regulation of adenylate cyclase activity involved in G-protein coupled receptor signaling pathway, positive regulation of glutamate secretion, positive regulation of acetylcholine secretion, neurotransmission, regulation of norepinephrine secretion, positive regulation of cAMP biosynthetic process, response to caffeine, positive regulation of synaptic transmission, GABAergic, synaptic transmission, glutamatergic, positive regulation of urine volume, positive regulation of renal sodium excretion, negative regulation of locomotion, vasodilation, response to drug, eating behavior, negative regulation of vascular permeability, negative regulation of cysteine-type endopeptidase activity involved in apoptotic process, response to alkaloid, negative regulation of neuron apoptotic process, positive regulation of circadian sleep/wake cycle, sleep, negative regulation of alpha-beta T cell activation, astrocyte activation, regulation of synaptic plasticity, neuron projection morphogenesis, positive regulation of protein secretion, negative regulation of inflammatory response, regulation of mitochondrial membrane potential, membrane depolarization, regulation of calcium ion transport, positive regulation of synaptic transmission, glutamatergic, excitatory postsynaptic potential, inhibitory postsynaptic potential, prepulse inhibition, positive regulation of apoptotic signaling pathway, |
| Ago2 | argonaute RISC catalytic subunit 2 | 15 D3  NC_000081.6 | transcription, DNA-templated, regulation of transcription, DNA-templated, regulation of translation, multicellular organism development, post-embryonic development, RNA secondary structure unwinding, miRNA metabolic process, positive regulation of gene expression, cell differentiation, gene silencing by RNA, pre-miRNA processing, siRNA loading onto RISC involved in RNA interference, production of miRNAs involved in gene silencing by miRNA, miRNA mediated inhibition of translation, mRNA cleavage involved in gene silencing by miRNA, miRNA loading onto RISC involved in gene silencing by miRNA, positive regulation of transcription from RNA polymerase II promoter, negative regulation of translational initiation, positive regulation of nuclear-transcribed mRNA poly(A) tail shortening, mRNA cleavage involved in gene silencing by siRNA, positive regulation of nuclear-transcribed mRNA catabolic process, deadenylation-dependent decay |
| Ankle1 | ankyrin repeat and LEM domain containing 1 | 8 B3.3  NC_000074.6 | DNA repair, protein export from nucleus, nuclear envelope organization, determination of adult lifespan, regulation of T cell differentiation in thymus, erythrocyte maturation, regulation of B cell differentiation, negative regulation of mitotic recombination, regulation of alpha-beta T cell differentiation, meiotic attachment of telomere to nuclear envelope, regulation of response to DNA damage stimulus, positive regulation of response to DNA damage stimulus, |
| Ankrd13d | ankyrin repeat domain 13 family, member D | 19 A  NC_000085.6 | [negative regulation of receptor internalization](http://amigo.geneontology.org/amigo/term/GO:0002091) |
| Ankrd22 | ankyrin repeat domain 22 | 19 C1  NC_000085.6 |  |
| Ap5b1 | adaptor-related protein complex 5, beta 1 subunit | 19 A  NC_000085.6 | protein transport, endosomal transport, |
| Apobec2 | apolipoprotein B mRNA editing enzyme, catalytic polypeptide 2 | 17 C  NC_000083.6 | [mRNA processing](http://www.ebi.ac.uk/QuickGO/GTerm?id=GO:0006397), [cytidine to uridine editing](http://www.ebi.ac.uk/QuickGO/GTerm?id=GO:0016554), [mRNA modification](http://www.ebi.ac.uk/QuickGO/GTerm?id=GO:0016556), [DNA demethylation](http://www.ebi.ac.uk/QuickGO/GTerm?id=GO:0080111), |
| Ar | androgen receptor | X C3  NC_000086.7 | in utero embryonic development, regulation of systemic arterial blood pressure, epithelial cell morphogenesis, transcription, DNA-templated, regulation of transcription, DNA-templated, regulation of transcription from RNA polymerase II promoter, spermatogenesis, single fertilization, copulation, male courtship behavior, positive regulation of cell proliferation, negative regulation of cell proliferation, male gonad development, fertilization, cellular process, regulation of gene expression, positive regulation of gene expression, skeletal muscle hypertrophy, male somatic sex determination, androgen receptor signaling pathway, intracellular receptor signaling pathway, positive regulation of intracellular estrogen receptor signaling pathway, Leydig cell differentiation, multicellular organism growth, positive regulation of phosphorylation, steroid hormone mediated signaling pathway, positive regulation of MAPK cascade, positive regulation of insulin-like growth factor receptor signaling pathway, single-organism reproductive behavior, positive regulation of cell differentiation, negative regulation of integrin biosynthetic process, positive regulation of integrin biosynthetic process, negative regulation of transcription, DNA-templated, positive regulation of transcription, DNA-templated, positive regulation of transcription from RNA polymerase II promoter, positive regulation of transcription from RNA polymerase III promoter, male sex differentiation, reproductive structure development, regulation of developmental growth, organ formation, male genitalia morphogenesis, negative regulation of epithelial cell proliferation, regulation of catalytic activity, positive regulation of NF-kappaB transcription factor activity, protein oligomerization, activation of prostate induction by androgen receptor signaling pathway, morphogenesis of an epithelial fold, lateral sprouting involved in mammary gland duct morphogenesis, regulation of prostatic bud formation, prostate gland growth, prostate gland epithelium morphogenesis, epithelial cell differentiation involved in prostate gland development, tertiary branching involved in mammary gland duct morphogenesis, mammary gland alveolus development, positive regulation of epithelial cell proliferation involved in prostate gland development, reproductive system development, seminiferous tubule development, regulation of establishment of protein localization to plasma membrane, negative regulation of extrinsic apoptotic signaling pathway, |
| AY074887 | cDNA sequence AY07488 | 9 B  NC_000075.6 | a[poptotic signaling pathway](http://amigo.geneontology.org/amigo/term/GO:0097190)  [cell cycle arrest](http://amigo.geneontology.org/amigo/term/GO:0007050)  [negative regulation of cell proliferation](http://amigo.geneontology.org/amigo/term/GO:0008285) |
| Bcl2l15 | BCLl2-like 15 | 3 F2.2  NC_000069.6 | [Regulation of Apoptosis by Parathyroid Hormone-related Protein](http://pathcards.genecards.org/card/regulation_of_apoptosis_by_parathyroid_hormone-related_protein). |
| Bora | bora, aurora kinase A activator | 14 E2.2  NC_000080.6 | [activation of protein kinase activity](http://amigo.geneontology.org/amigo/term/GO:0032147)  [regulation of mitotic nuclear division](http://amigo.geneontology.org/amigo/term/GO:0007088)  [regulation of mitotic nuclear division](http://amigo.geneontology.org/amigo/term/GO:0007088) |
| Btn2a2 | butyrophilin, subfamily 2, member A2 | 13 A3.1  NC_000079.6 | negative regulation of phosphatidylinositol 3-kinase signaling, negative regulation of cellular metabolic process, positive regulation of regulatory T cell differentiation, negative regulation of activated T cell proliferation, negative regulation of cytokine secretion, negative regulation of T cell receptor signaling pathway, negative regulation of protein kinase B signaling, negative regulation of ERK1 and ERK2 cascade, negative regulation of G1/S transition of mitotic cell cycle, |
| C1qtnf9 | C1q and tumor necrosis factor related protein 9 | 14 D1  NC_000080.6 | hormone activity,  Activates AMPK, AKT, and p44/42 MAPK signaling pathways |
| Cacna1e | calcium channel, voltage-dependent, R type, alpha 1E subunit | 1 G3  NC_000067.6 | behavioral fear response, regulation of heart rate, transport, ion transport, calcium ion transport, locomotory behavior, visual learning, transmission of nerve impulse, sensory perception of pain, sperm motility, regulation of ion transmembrane transport, glucose homeostasis, fear response, response to pain, behavioral response to pain, neurological system process, transmembrane transport, regulation of insulin secretion involved in cellular response to glucose stimulus, calcium ion import, calcium ion transmembrane transport, membrane depolarization during action potential, regulation of somatostatin secretion, |
| Calml3 | calmodulin-like 3 | 13 A1  NC_000079.6 | Activated PKN1 stimulates transcription of AR (androgen receptor) regulated genes KLK2 and KLK3 and Oxytocin signaling pathway, calcium ion binding.. |
| Caly | calcyon neuron-specific vesicular protein | 7 F4  NC_000073.6 | endocytosis, dopamine receptor signaling pathway, positive regulation of endocytosis, clathrin coat assembly, |
| Camkv | CaM kinase-like vesicle-associated | 9 F1  NC_000075.6 | protein phosphorylation, |
| Car13 | carbonic anhydrase 13 | 3 A1  NC_000069.6 | one-carbon metabolic process, |
| Cbx7 | chromobox 7 | 15 E1  NC_000081.6 | negative regulation of transcription from RNA polymerase II promoter, transcription, DNA-templated, regulation of transcription, DNA-templated, covalent chromatin modification, positive regulation of transcription elongation from RNA polymerase II promoter, response to drug, sebaceous gland development, |
| Ccdc78 | coiled-coil domain containing 78 | 17 A3.3  NC_000083.6 | skeletal muscle contraction, cell projection organization, de novo centriole assembly, |
| Ccdc87 | coiled-coil domain containing 8 | 7 A2  NC_000073.6 | microtubule binding |
| Cd79a | CD79A antigen (immunoglobulin-associated alpha) | 7 A3  NC_000073.6 | adaptive immune response, immune system process, cell surface receptor signaling pathway, B cell differentiation, B cell proliferation, B cell activation, B cell receptor signaling pathway. |
| Cdh13 | cadherin 13 | 8 E1  NC_000074.6 | mitotic cell cycle, positive regulation of endothelial cell proliferation, positive regulation of cell-matrix adhesion, sprouting angiogenesis, cell adhesion, homophilic cell adhesion via plasma membrane adhesion molecules, negative regulation of cell adhesion, Rho protein signal transduction, negative regulation of cell proliferation, calcium-dependent cell-cell adhesion via plasma membrane cell adhesion molecules, Rac protein signal transduction, lamellipodium assembly, regulation of endocytosis, positive regulation of cell migration, regulation of epidermal growth factor receptor signaling pathway, endothelial cell migration, keratinocyte proliferation, positive regulation of transcription from RNA polymerase II promoter, positive regulation of smooth muscle cell proliferation, positive regulation of calcium-mediated signaling, positive regulation of positive chemotaxis, localization within membrane, low-density lipoprotein particle mediated signaling, |
| Cdh8 | cadherin 8 | 8 D1  NC_000074.6 | cell adhesion, homophilic cell adhesion via plasma membrane adhesion molecules, chemical synaptic transmission, response to cold, synaptic transmission, glutamatergic, |
| Chodl | chondrolectin | 16 C3.1  NC_000082.6 | regulation of neuron projection development, |
| Chrna2 | cholinergic receptor, nicotinic, alpha polypeptide 2 (neuronal) | 14 D1  NC_000080.6 | ion transport, signal transduction, synaptic transmission, cholinergic, neuromuscular synaptic transmission, response to nicotine, protein heterooligomerization, cation transmembrane transport |
| Clcf1 | cardiotrophin-like cytokine factor 1 | 19 A  NC_000085.6 | cell surface receptor signaling pathway, multicellular organism development, positive regulation of cell proliferation, |
| Clcn1 | chloride channel, voltage-sensitive 1 | 6 B2.1  NC_000072.6 | ion transport, chloride transport, muscle contraction, neuronal action potential propagation, regulation of ion transmembrane transport, transmembrane transport, chloride transmembrane transport, |
| Cldn20 | claudin 20 | 17 A1  NC_000083.6 | Tight junction and Cell junction organization |
| Clmp | CXADR-like membrane protein | 9 A5.  NC_000075. | digestive tract development, |
| Col23a1 | collagen, type XXIII, alpha 1 | 11 B1.3  NC_000077.6 | collagen catabolic process, protein homotrimerization, |
| Coro6 | coronin 6 | 11 B5  NC_000077.6 | actin cytoskeleton organization, |
| Ctf2 | cardiotrophin 2 | 7 F3  NC_000073.6 | cell surface receptor signaling pathway, multicellular organism development, positive regulation of cell proliferation, |
| Crebbp | CREB binding protein | 16 A1  NC_000082.6 | negative regulation of transcription from RNA polymerase II promoter, transcription, DNA-templated, regulation of transcription, DNA-templated, protein acetylation, cell proliferation, positive regulation of gene expression, histone acetylation, N-terminal peptidyl-lysine acetylation, germ-line stem cell population maintenance, positive regulation of CREB transcription factor activity, cellular response to UV, cellular response to hepatocyte growth factor stimulus, positive regulation of transcription, DNA-templated, positive regulation of transcription from RNA polymerase II promoter, rhythmic process, positive regulation of sequence-specific DNA binding transcription factor activity, positive regulation of cell adhesion molecule production, cell-cell adhesion, positive regulation of G1/S transition of mitotic cell cycle, positive regulation of NIK/NF-kappaB signaling, |
| Cxcr6 | chemokine (C-X-C motif) receptor 6 | 9 F4  NC_000075.6 | chemotaxis, inflammatory response, signal transduction, G-protein coupled receptor signaling pathway, |
| Cyp8b1 | cytochrome P450, family 8, subfamily b, polypeptide 1 | 9 F4  NC_000075.6 | oxidation-reduction process, |
| Cyyr1 | cysteine and tyrosine-rich protein 1 | 16 C3.3  NC_000082.6 | No biological process available |
| Degs2 | delta(4)-desaturase, sphingolipid 2 | 12 F1  NC_000078.6 | lipid metabolic process, sphinganine metabolic process, sphingolipid biosynthetic process, ceramide biosynthetic process, oxidation-reduction process, |
| Dgki | diacylglycerol kinase, iota | 6 B1  NC_000072.6 | protein kinase C-activating G-protein coupled receptor signaling pathway, neurotransmitter secretion, intracellular signal transduction, regulation of GTPase activity, positive regulation of Ras protein signal transduction, lipid phosphorylation, habituation, regulation of synaptic transmission, glutamatergic, excitatory postsynaptic potential, regulation of long term synaptic depression, |
| Dock3 | dedicator of cyto-kinesis | 9 F1  NC_000075.6 | small GTPase mediated signal transduction, |
| Dstyk | dual serine/threonine and tyrosine protein kinase | 1 E4  NC_000067.6 | protein phosphorylation, phosphorylation, positive regulation of kinase activity, intracellular signal transduction, cellular response to fibroblast growth factor stimulus, positive regulation of fibroblast growth factor receptor signaling pathway, positive regulation of ERK1 and ERK2 cascade, |
| Dusp4 | dual specificity phosphatase 4 | 8 A4  NC_000074.6 | inactivation of MAPK activity, endoderm formation, protein dephosphorylation, dephosphorylation, peptidyl-tyrosine dephosphorylation, peptidyl-threonine dephosphorylation, negative regulation of ERK1 and ERK2 cascade |
| Eng | endoglin | 2 B  NC_000068.7 | chronological cell aging, angiogenesis, patterning of blood vessels, vasculogenesis, positive regulation of protein phosphorylation, heart looping, sprouting angiogenesis, outflow tract septum morphogenesis, epithelial to mesenchymal transition involved in endocardial cushion formation, endocardial cushion morphogenesis, cardiac ventricle morphogenesis, cardiac atrium morphogenesis, ventricular trabecula myocardium morphogenesis, cell migration involved in endocardial cushion formation, regulation of transcription, DNA-templated, cell adhesion, transforming growth factor beta receptor signaling pathway, heart development, positive regulation of gene expression, negative regulation of gene expression, regulation of cardiac muscle cell apoptotic process, positive regulation of pathway-restricted SMAD protein phosphorylation, regulation of transforming growth factor beta receptor signaling pathway, central nervous system vasculogenesis, negative regulation of cell migration, positive regulation of BMP signaling pathway, negative regulation of protein autophosphorylation, response to corticosteroid, positive regulation of collagen biosynthetic process, dorsal aorta morphogenesis, positive regulation of angiogenesis, positive regulation of transcription from RNA polymerase II promoter, smooth muscle tissue development, artery morphogenesis, venous blood vessel morphogenesis, atrial cardiac muscle tissue morphogenesis, bone development, extracellular matrix constituent secretion, cellular response to mechanical stimulus, endocardial cushion to mesenchymal transition, vascular smooth muscle cell development, positive regulation of epithelial to mesenchymal transition involved in endocardial cushion formation, positive regulation of vascular smooth muscle cell differentiation, cardiac jelly development, atrioventricular canal morphogenesis, regulation of cell proliferation involved in heart morphogenesis. |
| \| Fam126a \| \| --- \| | family with sequence similarity 126, member A | 5 A3  NC_000071.6 | myelination, phosphatidylinositol phosphorylation, establishment of protein localization to plasma membrane, |
| Fam131b | family with sequence similarity 131, member B | 6 B2.1  NC_000072.6 | positive regulation of defense response to virus by host, mitophagy in response to mitochondrial depolarization, xenophagy |
| \| Fam217b \| \| --- \| | family with sequence similarity 217, member B | 2 H4  NC_000068.7 | No biological process available |
| \| Fam71e2 \| \| --- \| | family with sequence similarity 71, member E2 | 7 A1  NC_000073.6 | No biological process available |
| Fcgr2b | Fc receptor, IgG, low affinity IIb | 1 H3  NC_000067.6 | negative regulation of type I hypersensitivity, immune complex clearance, negative regulation of immunoglobulin production, negative regulation of inflammatory response to antigenic stimulus, negative regulation of acute inflammatory response to antigenic stimulus, negative regulation of humoral immune response mediated by circulating immunoglobulin, receptor-mediated endocytosis, phagocytosis, engulfment, defense response, immune response, cell surface receptor signaling pathway, response to bacterium, immunoglobulin mediated immune response, antigen processing and presentation of exogenous peptide antigen via MHC class II, negative regulation of B cell proliferation, endocytic recycling, negative regulation of interleukin-10 production, negative regulation of neutrophil apoptotic process, mast cell activation, negative regulation of phagocytosis, positive regulation of phagocytosis, negative regulation of immune response, cellular response to molecule of bacterial origin, |
| Frrs1l | ferric-chelate reductase 1 like | 4 B3  NC_000070.6 | encodes a component of the outer-core of an alpha-amino-3-hydroxy-5-methyl-4-isoxazolepropionic acid (AMPA) receptor protein in the brain. The encoded protein is thought to interact with inner-core components of the receptor, and play a role in the modulation of glutamate signaling. |
| Gabbr2 | gamma-aminobutyric acid (GABA) B receptor, 2 | 4 B1  NC_000070.6 | signal transduction, G-protein coupled receptor signaling pathway, gamma-aminobutyric acid signaling pathway, |
| Gcnt7 | glucosaminyl (N-acetyl) transferase family member 7 | 2 H3  NC_000068.7 | protein glycosylation, |
| Gfra3 | glial cell line derived neurotrophic factor family receptor alpha 3 | 18; 18 B1  NC_000084.6 | neuron migration, transmembrane receptor protein tyrosine kinase signaling pathway, axon guidance, sympathetic nervous system development, neuron development, |
| Gjb4 | gap junction protein, beta 4 | 4 D2.2  NC_000070 | cell communication, sensory perception of smell, olfactory behavior, |
| Gm4858 | TD and POZ domain containing 8 | 3 F1  NC_000069.6 | regulation of proteolysis, protein ubiquitination involved in ubiquitin-dependent protein catabolic process, proteasome-mediated ubiquitin-dependent protein catabolic process, |
| Gm5617 | predicted gene 5617 | 9 A5.3  NC_000075.6 | No biological data availabe |
| Gpr135 | G protein-coupled receptor 135 | 12 C3  NC_000078.6 | signal transduction, G-protein coupled receptor signaling pathway, |
| Gpr137c | G protein-coupled receptor 137C | 14 C1  NC_000080.6 | No biological data availabe |
| Gpr171 | G protein-coupled receptor 171 | 3 D  NC_000069 | signal transduction, G-protein coupled receptor signaling pathway, negative regulation of myeloid cell differentiation |
| H2-T24 | histocompatibility 2, T region locus 24 | 17 B1  NC_000083.6 | antigen processing and presentation of peptide antigen via MHC class I, |
| Hpca | hippocalcin | 4 D2.2  NC_000070.6 | brain development, positive regulation of phospholipase activity, calcium-mediated signaling, negative regulation of guanylate cyclase activity, activation of phospholipase D activity, positive regulation of adenylate cyclase activity, inner ear development, retina development in camera-type eye, cellular response to electrical stimulus, positive regulation of protein targeting to membrane, response to ketamine, response to L-glutamate, cellular response to monosodium glutamate, response to Aroclor 1254, |
| Hsd3b7 | hydroxy-delta-5-steroid dehydrogenase, 3 beta- and steroid delta-isomerase 7 | 7 F3  NC_000073.6 | regulation of cell growth, steroid biosynthetic process, cholesterol catabolic process, B cell chemotaxis, oxidation-reduction process |
| Ikzf3 | IKAROS family zinc finger 3 | 11 D  NC_000077.6 | transcription, DNA-templated, regulation of transcription, DNA-templated, regulation of B cell proliferation, B cell activation, regulation of apoptotic process, regulation of B cell differentiation, regulation of lymphocyte differentiation, positive regulation of transcription from RNA polymerase II promoter, |
| Il15 | interleukin 15 | 8 C2  NC_000074.6 | natural killer cell differentiation, NK T cell proliferation, inflammatory response, immune response, signal transduction, aging, positive regulation of cell proliferation, skeletal muscle atrophy, hyaluronan metabolic process, positive regulation of interleukin-17 production, positive regulation of natural killer cell proliferation, positive regulation of natural killer cell differentiation, positive regulation of T cell proliferation, tyrosine phosphorylation of Stat5 protein, positive regulation of tyrosine phosphorylation of Stat3 protein, extrathymic T cell selection, regulation of T cell differentiation, cell maturation, lymph node development, negative regulation of smooth muscle cell proliferation, regulation of defense response to virus by host, positive regulation of immune response, cellular response to vitamin D, positive regulation of protein O-linked glycosylation, |
| Il1a | interleukin 1 alpha | 2 F1  NC_000068.7 | fever generation, connective tissue replacement involved in inflammatory response wound healing, inflammatory response, immune response, negative regulation of cell proliferation, positive regulation of vascular endothelial growth factor production, positive regulation of gene expression, positive regulation of steroid biosynthetic process, cytokine-mediated signaling pathway, keratinization, positive regulation of prostaglandin secretion, positive regulation of interleukin-6 production, positive regulation of stress-activated MAPK cascade, regulation of actin cytoskeleton organization, cellular response to heat, ectopic germ cell programmed cell death, positive regulation of I-kappaB kinase/NF-kappaB signaling, positive regulation of interleukin-2 biosynthetic process, positive regulation of angiogenesis, positive regulation of mitotic nuclear division, positive regulation of transcription from RNA polymerase II promoter, positive regulation of JNK cascade, response to copper ion, positive regulation of protein secretion, positive regulation of cytokine secretion, positive regulation of cell division, regulation of sensory perception of pain, positive regulation of ERK1 and ERK2 cascade, interleukin-1-mediated signaling pathway, positive regulation of monocyte chemotactic protein-1 production, extrinsic apoptotic signaling pathway in absence of ligand, positive regulation of neutrophil migration, negative regulation of establishment of Sertoli cell barrier, positive regulation of neutrophil extravasation, |
| Impg1 | interphotoreceptor matrix proteoglycan 1 | 9 E1  NC_000075.6 | encodes a protein that is a major component of the retinal interphotoreceptor matrix. The encoded protein is a proteoglycan that is thought to play a role in maintaining viability of photoreceptor cells and in adhesion of the neural retina to the retinal pigment epithelium |
| Inpp5f | inositol polyphosphate-5-phosphatase F | 7 F3  NC_000073.6 | positive regulation of receptor recycling, adult locomotory behavior, cardiac muscle hypertrophy in response to stress, phosphatidylinositol catabolic process, negative regulation of peptidyl-serine phosphorylation, negative regulation of tyrosine phosphorylation of Stat3 protein, phosphatidylinositol dephosphorylation, phosphatidylinositol-mediated signaling, negative regulation of axon regeneration, regulation of protein kinase B signaling, vacuolar proton-transporting V-type ATPase complex assembly, clathrin-mediated endocytosis, regulation of cell motility, regulation of endocytic recycling, |
| Jag2 | jagged 2 | 12 F1  NC_000078.6 | skeletal system development, in utero embryonic development, cell fate determination, respiratory system process, cell communication, Notch signaling pathway, multicellular organism development, spermatogenesis, sensory perception of sound, auditory receptor cell fate commitment, morphogenesis of embryonic epithelium, cell differentiation, regulation of cell adhesion, T cell differentiation, regulation of cell proliferation, odontogenesis of dentin-containing tooth, gamma-delta T cell differentiation, thymic T cell selection, positive regulation of Notch signaling pathway, epithelial cell apoptotic process involved in palatal shelf morphogenesis, |
| Kcng3 | potassium voltage-gated channel, subfamily G, member 3 | 17 E4  NC_000083.6 | transport, ion transport, potassium ion transport, regulation of ion transmembrane transport, protein homooligomerization, transmembrane transport, potassium ion transmembrane transport, |
| Kcnj4 | potassium inwardly-rectifying channel, subfamily J, member 4 | 15 E1  NC_000081.6 | transport, ion transport, potassium ion transport, potassium ion import, regulation of ion transmembrane transport |
| Kctd4 | potassium channel tetramerisation domain containing 4 | 14 D3  NC_000080.6 | Protein homooligomerization, |
| Kdm4d | lysine (K)-specific demethylase 4D | 9 A2  NC_000075.6 | double-strand break repair via homologous recombination, regulation of protein phosphorylation, transcription, DNA-templated, regulation of transcription, DNA-templated, cellular response to DNA damage stimulus, covalent chromatin modification, histone H3-K9 demethylation, positive regulation of chromatin binding, oxidation-reduction process, cellular response to ionizing radiation, negative regulation of histone H3-K9 trimethylation, positive regulation of double-strand break repair via nonhomologous end joining, |
| Kif13b | kinesin family member 13B | 14 D1  NC_000080.6 | microtubule-based movement, cytoskeleton-dependent intracellular transport, regulation of axonogenesis, |
| Krr1 | KRR1, small subunit (SSU) processome component, homolog (yeast) | 10 D2  NC_000076.6 | maturation of SSU-rRNA from tricistronic rRNA transcript (SSU-rRNA, 5.8S rRNA, LSU-rRNA), rRNA processing, ribosome biogenesis, |
| Krt80 | keratin 80 | 15 F2  NC_000081.6 | Keratins are intermediate filament proteins responsible for the structural integrity of epithelial cells and are subdivided into epithelial keratins and hair keratins. |
| Lgi4 | leucine-rich repeat LGI family, member 4 | 7 B1  NC_000073.6 | Adult locomotory behavior, glial cell proliferation, Schwann cell development, glial cell development, myelination in peripheral nervous system, gliogenesis, neuron maturation, myelination, |
| Lman1l | lectin, mannose-binding 1 like | 9 B  NC_000075.5 | ER to Golgi vesicle-mediated transport, endoplasmic reticulum organization, Golgi organization, |
| Lnpep | leucyl/cystinyl aminopeptidase | 17 A3.2  NC_000083.6 | proteolysis, cell-cell signaling, regulation of blood pressure, protein catabolic process, peptide catabolic process, SMAD protein signal transduction, membrane organization, |
| Lrp1b | low density lipoprotein-related protein 1B | 2 B  NC_000068.7 | utero embryonic development, endocytosis, |
| Lrrc75a | leucine rich repeat containing 75A | 11 B2  NC_000077.6 | No biological data available |
| Mcoln3 | mucolipin 3 | 3 H2  NC_000069.6 | transport, ion transport, sensory perception of sound, locomotory behavior, auditory receptor cell differentiation, |
| Mettl1 | methyltransferase like 1 | 10 D3  NC_000076.6 | tRNA modification, tRNA processing, methylation, RNA (guanine-N7)-methylation, |
| \| Mettl11b \| \| --- \| | methyltransferase like 11B | 1 H2.1  NC_000067.6 | protein methylation, N-terminal protein amino acid methylation, methylation, |
| Mfsd9 | major facilitator superfamily domain containing 9 | 1 B  NC_000067.6 | transport, transmembrane transport, |
| Mmp20 | matrix metallopeptidase 20 (enamelysin | 9 A1  NC_000075.6 | proteolysis, extracellular matrix disassembly, protein catabolic process, amelogenesis, |
| Morn3 | MORN repeat containing 3 | 5 F  NC_000071.6 | Regulation of spermiogenesis |
| Msi1 | musashi RNA-binding protein 1 | 5 F  NC_000071.6 | response to hormone, |
| Myo7a | myosin VIIA | 7 E1  NC_000073.6 | phagolysosome assembly, transport, intracellular protein transport, phagocytosis, lysosome organization, sensory perception, visual perception, sensory perception of sound, cell projection organization, actin filament-based movement, inner ear morphogenesis, mechanoreceptor differentiation, auditory receptor cell differentiation, post-embryonic organ morphogenesis, inner ear development, sensory perception of light stimulus, equilibrioception, pigment granule localization, pigment granule transport, auditory receptor cell stereocilium organization, inner ear receptor cell differentiation, inner ear receptor stereocilium organization, |
| \| Naaladl1 \| \| --- \| | N-acetylated alpha-linked acidic dipeptidase-like 1 | 19 A  NC_000085.6 | proteolysis, metabolic process, |
| Nav3 | neuron navigator 3 | 10 D1  NC_000076.6 | Prorneuron regeneration |
| Nos1 | nitric oxide synthase 1, neuronal | 5 F  NC_000071.6 | response to hypoxia, regulation of sodium ion transport, arginine catabolic process, nitric oxide biosynthetic process, striated muscle contraction, nitric oxide mediated signal transduction, regulation of heart contraction, negative regulation of cell proliferation, response to heat, peptidyl-cysteine S-nitrosylation, positive regulation of guanylate cyclase activity, negative regulation of peptidyl-serine phosphorylation, multicellular organismal response to stress, positive regulation of histone acetylation, exogenous drug catabolic process, negative regulation of apoptotic process, negative regulation of potassium ion transport, response to peptide hormone, response to estrogen, negative regulation of blood pressure, negative regulation of heart contraction, positive regulation of transcription, DNA-templated, negative regulation of vasoconstriction, positive regulation of vasodilation, positive regulation of transcription from RNA polymerase II promoter, negative regulation of insulin secretion, behavioral response to cocaine, regulation of neurogenesis, negative regulation of hydrolase activity, negative regulation of cytosolic calcium ion concentration, negative regulation of serotonin uptake, negative regulation of calcium ion transport, regulation of sensory perception of pain, oxidation-reduction process, cellular response to mechanical stimulus, cellular response to growth factor stimulus, cellular response to epinephrine stimulus, positive regulation of the force of heart contraction, positive regulation of long-term synaptic potentiation, positive regulation of adrenergic receptor signaling pathway involved in heart process, positive regulation of neuron death, positive regulation of sodium ion transmembrane transport, |
| Npsr1 | neuropeptide S receptor 1 | 9 A3-A4  NC_000075.6 | signal transduction, G-protein coupled receptor signaling pathway, neuropeptide signaling pathway, eating behavior, positive regulation of release of sequestered calcium ion into cytosol, righting reflex, positive regulation of ERK1 and ERK2 cascade, negative regulation of eating behavior, negative regulation of defecation, |
| Ntm | neurotrimin | 9 A4  NC_000075.6 | response to hypoxia, regulation of sodium ion transport, arginine catabolic process, nitric oxide biosynthetic process, striated muscle contraction, nitric oxide mediated signal transduction, regulation of heart contraction, negative regulation of cell proliferation, response to heat, peptidyl-cysteine S-nitrosylation, positive regulation of guanylate cyclase activity, negative regulation of peptidyl-serine phosphorylation, multicellular organismal response to stress, positive regulation of histone acetylation, exogenous drug catabolic process, negative regulation of apoptotic process, negative regulation of potassium ion transport, response to peptide hormone, response to estrogen, negative regulation of blood pressure, negative regulation of heart contraction, positive regulation of transcription, DNA-templated, negative regulation of vasoconstriction, positive regulation of vasodilation, positive regulation of transcription from RNA polymerase II promoter, negative regulation of insulin secretion, behavioral response to cocaine, regulation of neurogenesis, negative regulation of hydrolase activity, negative regulation of cytosolic calcium ion concentration, negative regulation of serotonin uptake, negative regulation of calcium ion transport, regulation of sensory perception of pain, oxidation-reduction process, cellular response to mechanical stimulus, cellular response to growth factor stimulus, cellular response to epinephrine stimulus, positive regulation of the force of heart contraction, positive regulation of long-term synaptic potentiation, positive regulation of adrenergic receptor signaling pathway involved in heart process, positive regulation of neuron death, positive regulation of sodium ion transmembrane transport |
| \| Olfm2 \| \| --- \| | olfactomedin 2 | 9 A3  NC_000075.6 | visual perception, locomotory behavior, protein secretion, positive regulation of smooth muscle cell differentiation |
| \| Olfr1137 \| \| --- \| | olfactory receptor 1137 | 2 D  NC_000068.7 | G-protein coupled receptor signaling pathway, sensory perception of smell, |
| \| Olfr1138 \| \| --- \| | olfactory receptor 1138 | 2 D  NC_000068.7 | G-protein coupled receptor signaling pathway, sensory perception of smell |
| Olig2 | oligodendrocyte transcription factor 2 | 16 C3.3  NC_000082.6 | negative regulation of transcription from RNA polymerase II promoter, transcription, DNA-templated, regulation of transcription, DNA-templated, multicellular organism development, nervous system development, spinal cord motor neuron differentiation, spinal cord oligodendrocyte cell differentiation, spinal cord oligodendrocyte cell fate specification, oligodendrocyte cell fate specification, thalamus development, neuron differentiation, myelination, negative regulation of neuron differentiation, neuron fate commitment, oligodendrocyte differentiation, positive regulation of oligodendrocyte differentiation, |
| Otud6a | OTU domain containing 6A | X C3  NC_000086. | proteolysis, protein K29-linked deubiquitination, protein K11-linked deubiquitination, protein K27-linked deubiquitination, protein K33-linked deubiquitination. |
| Papln | papilin, proteoglycan-like sulfated glycoprotein | 12 D1  NC_000078.6 | negative regulation of peptidase activity, |
| Pax7 | paired box 7 | 4 D3  NC_000070.6 | chromatin remodeling, transcription, DNA-templated, regulation of transcription, DNA-templated, multicellular organism development, muscle organ development, skeletal muscle tissue development, organ morphogenesis, regulation of cell fate commitment, regulation of gene expression, skeletal muscle satellite cell commitment, spinal cord association neuron differentiation, dorsal/ventral neural tube patterning, positive regulation of histone methylation, regulation of protein binding, skeletal muscle tissue regeneration, positive regulation of transcription from RNA polymerase II promoter, neuron fate commitment, embryonic skeletal system development, cartilage development, muscle tissue morphogenesis, positive regulation of myoblast proliferation, |
| Pcp2 | Purkinje cell protein 2 (L7) | 8 A1.1  NC_000074.6 | rhodopsin mediated signaling pathway, |
| \| Pdcd1lg2 \| \| --- \| | programmed cell death 1 ligand 2 | 19 C1  NC_000085.6 | negative regulation of interferon-gamma production, negative regulation of interleukin-10 production, positive regulation of T cell proliferation, negative regulation of T cell proliferation, negative regulation of activated T cell proliferation, |
| Pgp | phosphoglycolate phosphatase | 17 A3.3  NC_000083.6 | carbohydrate metabolic process, glycerol biosynthetic process, glycerophospholipid metabolic process, metabolic process, dephosphorylation, peptidyl-tyrosine dephosphorylation, negative regulation of gluconeogenesis |
| \| Pgpep1l \| \| --- \| | pyroglutamyl-peptidase I-like | 7 C  NC_000073.6 | proteolysis, |
| Pik3ap1 | phosphoinositide-3-kinase adaptor protein 1 | 19 C3  NC_000085.6 | positive regulation of phosphatidylinositol 3-kinase signaling, negative regulation of toll-like receptor signaling pathway, positive regulation of toll-like receptor signaling pathway, toll-like receptor 2 signaling pathway, toll-like receptor 4 signaling pathway, toll-like receptor 7 signaling pathway, toll-like receptor 9 signaling pathway, regulation of I-kappaB kinase/NF-kappaB signaling, regulation of MAPK cascade, regulation of inflammatory response |
| Pou6f2 | POU domain, class 6, transcription factor 2 | 13 A2  NC_000079.6 | transcription, DNA-templated, regulation of transcription, DNA-templated, transcription from RNA polymerase II promoter, multicellular organism development, |
| Prorsd1 | prolyl-tRNA synthetase domain containing 1 | 11 A3.3  NC_000077.6 | No biological data available |
| Psg25 | pregnancy-specific glycoprotein 25 | 7 A3  NC_000073.6 | female pregnancy, |
| Pura | purine rich element binding protein A | 18 B2  NC_000084.6 | DNA unwinding involved in DNA replication, transcription, DNA-templated, regulation of transcription, DNA-templated, apoptotic process, mitotic cell cycle checkpoint, nervous system development, cell proliferation, positive regulation of cell proliferation, cell differentiation, regulation of cell proliferation, negative regulation of transcription, DNA-templated |
| Rcvrn | recoverin | 11 B3  NC_000077.6 | visual perception, phototransduction, response to stimulus, regulation of calcium ion transport, |
| Rims3 | regulating synaptic membrane exocytosis 3 | 4 D2.2  NC_000070.6 | transport, neurotransmitter transport, exocytosis, calcium ion regulated exocytosis, regulation of membrane potential, calcium ion-regulated exocytosis of neurotransmitter, mitophagy in response to mitochondrial depolarization, regulation of synaptic vesicle exocytosis |
| Rnf125 | ring finger protein 125 | 18 A2  NC_000084.6 | protein polyubiquitination, adaptive immune response, immune system process, protein ubiquitination, positive regulation of proteasomal ubiquitin-dependent protein catabolic process, |
| S100a7a | S100 calcium binding protein A7A | 3 F1  NC_000069.6 | inflammatory response, cytokine metabolic process, |
| Samd5 | sterile alpha motif domain containing 5 | 10 A1  NC_000076.6 | No biological data available |
| Sdr9c7 | 4short chain dehydrogenase/reductase family 9C, member 7 | 10 D3  NC_000076.6 | oxidation-reduction process, |
| Slc46a3 | solute carrier family 46, member 3 | 5 G3  NC_000071.6 | transmembrane transport, |
| Slc9b1 | solute carrier family 9, subfamily B (NHA1, cation proton antiporter 1), member 1 | 3 G3  NC_000069.6 | transport, cation transport, transmembrane transport, |
| Spata17 | spermatogenesis associated 17 | 1 H5  NC_000067.6 | apoptosis |
| Spata25 | spermatogenesis associated 25 | 2 H3  NC_000068.7 | spermatogenesis, cell differentiation, |
| Spidr | scaffolding protein involved in DNA repair | 16 A2  NC_000082.6 | double-strand break repair via homologous recombination, DNA repair, DNA recombination, cellular response to DNA damage stimulus, regulation of double-strand break repair via homologous recombination, positive regulation of protein complex assembly, regulation of establishment of protein localization to chromosome, cellular response to ionizing radiation, cellular response to hydroxyurea, cellular response to camptothecin, positive regulation of double-strand break repair, |
| St8sia6 | ST8 alpha-N-acetyl-neuraminide alpha-2,8-sialyltransferase 6 | 2 A1  NC_000068.7 | ganglioside biosynthetic process, protein glycosylation, protein O-linked glycosylation, glycoprotein metabolic process, glycolipid biosynthetic process, oligosaccharide metabolic process, carbohydrate biosynthetic process. |
| Susd3 | sushi domain containing 3 | 13 A5  NC_000079.6 | No biological data available |
| Syde1 | synapse defective 1, Rho GTPase, homolog 1 (C. elegans) | 10 C1  NC_000076.6 | synaptonemal complex assembly, signal transduction, synaptic vesicle docking, positive regulation of synaptic transmission, activation of GTPase activity, |
| Sync | syncoilin | 4 D2.2  NC_000070.6 | intermediate filament-based process, |
| Sypl2 | synaptophysin-like 2 | 3 F2.3  NC_000069.6 | transport, cellular calcium ion homeostasis, substantia nigra development, |
| Syt17 | synaptotagmin XVII | 7 F1  NC_000073.6 | exocytosis, vesicle fusion, regulation of calcium ion-dependent exocytosis, cell differentiation, calcium ion-regulated exocytosis of neurotransmitter, positive regulation of dendrite extension, |
| Tbl1x | transducin (beta)-like 1 X-linked | X B  NC_000086.7 | negative regulation of transcription from RNA polymerase II promoter, transcription, DNA-templated, regulation of transcription, DNA-templated, regulation of transcription from RNA polymerase II promoter, proteolysis, sensory perception of sound, histone deacetylation, proteasome-mediated ubiquitin-dependent protein catabolic process, response to estrogen, fat cell differentiation, negative regulation of transcription, DNA-templated, positive regulation of transcription, DNA-templated, positive regulation of transcription from RNA polymerase II promoter, response to steroid hormone, canonical Wnt signaling pathway, |
| Tbx6 | T-box 6 | 7 F3  NC_000073.6 | negative regulation of transcription from RNA polymerase II promoter, mesoderm formation, cell fate specification, transcription, DNA-templated, regulation of transcription, DNA-templated, multicellular organism development, negative regulation of neuron projection development, negative regulation of neuron maturation, signal transduction involved in regulation of gene expression, somite rostral/caudal axis specification, positive regulation of transcription from RNA polymerase II promoter, |
| Tcte1 | t-complex-associated testis expressed | 17 B3  NC_000083.6 | No biological data available |
| Tex101 | testis expressed gene 101 | 7 A3  NC_000073.6 | positive regulation of leukocyte activation, positive regulation of peptidyl-tyrosine phosphorylation, positive regulation of release of sequestered calcium ion into cytosol, |
| Tmem200b | transmembrane protein 200B | 4 D2.3  NC_000070.6 | No biological data available |
| Tmem63c | transmembrane protein 63c | 12 D2  NC_000078.6 | ion transport, cation transport, |
| Tmod4 | tropomodulin 4 | 3 F2.1  NC_000069.6 | muscle contraction, actin filament organization, myofibril assembly, pointed-end actin filament capping, |
| Tnfrsf13b | tumor necrosis factor receptor superfamily, member 13b | 11 B2  NC_000077.6 | B cell homeostasis, hematopoietic progenitor cell differentiation, adaptive immune response, immune system process, cell surface receptor signaling pathway, negative regulation of B cell proliferation, |
| Tnfrsf25 | tumor necrosis factor receptor superfamily, member 25 | 4 E2  NC_000070.6 | inflammatory response, immune response, signal transduction, multicellular organism development, response to lipopolysaccharide, regulation of cell proliferation, regulation of apoptotic process, positive regulation of MAPK cascade, apoptotic signaling pathway, |
| Trhr2 | thyrotropin releasing hormone receptor | 15 B3.2  NC_000081.6 | phospholipase C-activating G-protein coupled receptor signaling pathway |
| \| Ttc9 \| \| --- \| | tetratricopeptide repeat domain 9 | 12 D1  NC_000078.6 | chaperone-mediated protein folding, |
| Ttyh1 | tweety family member 1 | 7 A1  NC_000073.6 | transport, ion transport, chloride transport, mitotic nuclear division, cell adhesion, single organismal cell-cell adhesion, cell-substrate adhesion, regulation of anion transport, filopodium assembly, chloride transmembrane transport, |
| Usp50 | ubiquitin specific peptidase 50 | 2 F1  NC_000068.7 | transcription from RNA polymerase I promoter, transcription from RNA polymerase II promoter, transcription from RNA polymerase III promoter, proteolysis, ubiquitin-dependent protein catabolic process, protein deubiquitination, |
| Vwa5b1 | von Willebrand factor A domain containing 5B1 | 4 D3  NC_000070.6 | No biological data available |
| \| Vwa5b2 \| \| --- \| | von Willebrand factor A domain containing 5B2 | 16 B1  NC_000082.6 | No biological data available |
| Zbtb8os | zinc finger and BTB domain containing 8 opposite strand | 4 D2.2  NC_000070.6 | tRNA splicing, via endonucleolytic cleavage and ligation, tRNA processing, |
| \| Zc2hc1a \| \| --- \| | zinc finger, C2HC-type containing 1A | 3 A1  NC_000069.6 | No biological data available |
| Zfp128 | zinc finger protein 128 | 7 A1  NC_000073.6 | negative regulation of transcription from RNA polymerase II promoter, transcription, DNA-templated, regulation of transcription, DNA-templated, BMP signaling pathway |
| Zfp382 | zinc finger protein 382 | 7 B1  NC_000073.6 | negative regulation of transcription from RNA polymerase II promoter, transcription, DNA-templated, regulation of transcription, DNA-templated, negative regulation of transcription, DNA-templated, positive regulation of transcription from RNA polymerase II promoter, |
| Zfp605 | zinc finger protein 605 | 5 F  NC_000071.6 | regulation of transcription, DNA-templated, |
| Zfpm1 | zinc finger protein, multitype 1 | 8 E1  NC_000074.6 | negative regulation of transcription from RNA polymerase II promoter, outflow tract morphogenesis, atrioventricular valve morphogenesis, mitral valve formation, tricuspid valve formation, transcription, DNA-templated, regulation of transcription, DNA-templated, heart development, regulation of definitive erythrocyte differentiation, erythrocyte differentiation, megakaryocyte differentiation, platelet formation, granulocyte differentiation, negative regulation of protein binding, regulation of chemokine production, embryonic hemopoiesis, megakaryocyte development, positive regulation of interferon-gamma biosynthetic process, negative regulation of interleukin-4 biosynthetic process, negative regulation of fat cell differentiation, positive regulation of transcription from RNA polymerase II promoter, homeostasis of number of cells, cardiac muscle tissue morphogenesis, definitive erythrocyte differentiation, primitive erythrocyte differentiation, negative regulation of mast cell differentiation, ventricular septum morphogenesis, atrial septum morphogenesis, transcriptional activation by promoter-enhancer looping, |
